# Training needs assessment tools for the public health workforce at an institutional and individual level: a review

**DOI:** 10.1093/eurpub/ckad183

**Published:** 2023-10-25

**Authors:** Katarzyna Czabanowska, Pablo Rodriguez Feria

**Affiliations:** Department of International Health, Care and Public Health Research Institute CAPHRI, FHML, Maastricht University, Maastricht, the Netherlands; Department of Health Policy Management, Institute of Public Health, Jagiellonian University, Krakow, Poland; Department of International Health, Care and Public Health Research Institute CAPHRI, FHML, Maastricht University, Maastricht, the Netherlands; Departamento de Salud Pública, Facultad de Medicina, Universidad de Los Andes, Bogota, Colombia

## Abstract

**Background:**

The public health workforce (PHW) needs to have the necessary capacities to provide healthcare services and public health services. Training needs assessments (TNA) is necessary to assess and understand PHW and their capacities to provide services. This review attempts to identify and describe published studies on tools and methodologies for TNA of the workforce used in public health and health-related fields.

**Methods:**

A systematized review of literature was carried out in February 2022. Cochrane Handbook for systematic review version 5.2.0 and PRISMA 2020 statement were used to guide reporting. This review includes original research, reports and grey literature from the websites of public health organizations in English.

**Results:**

This review included 38 documents for evidence synthesis. Twenty-seven documents were indexed literature (71%) and 11 were grey documents (29%). TNA documents were published between 1999 and 2022. TNA was performed in many countries around the world. The organizations used either a validated questionnaire or created their own tools to perform organizational and individual self-assessments. The TNA tools were developed using different methods such as expert panels, literature reviews, stakeholder interviews and quantitative surveys.

**Conclusion:**

TNA is useful for defining and characterizing the public health workforce in every organization. Workforces consist of individuals who have their own training needs to fulfill their tasks. Therefore, individual and organizational TNA should be combined to study the public health workforce and their capacities.

## Introduction

Assessing and understanding public health workforce capacities is a prerequisite to prepare both organizations and the workforce to effectively address, prepare for and respond to health challenges. ‘A capacity assessment identifies capacity on three levels: individual, organizational and enabling environment’^1^ (Supplementary data 1). It also identifies the relations between these levels to identify gaps in capacities, competencies and know-how.^2^ The workforces share organization’s mission, vision and objectives; however, they consist of individuals who have their own training needs to fulfill their tasks. Therefore, individual and organizational training needs assessments should be combined to study the public health workforce and their capacities with the aim of improving public health systems’ efficiency. While individual assessment provides information on which employees need training and what kind of training, the organizational assessment looks at organizational performance and competencies which are needed to deliver public health services in line with the mission and strategy of the organization.^3^ More specifically, both types of assessment evaluate the internal environment to establish the differences between the existing and required capacities in order to identify deficits and define the most optimal organizational performance considering processes and environmental and systemic disruptions.^4^ Therefore, the public health workforce needs to be assessed first to match their needs with continuing post-graduate competency-based and timely training. Training needs assessment (TNA) ‘looks at the skills, knowledge and attitudes of potential trainees and uses this information to determine if and how the issue can be improved by training’.^1^ To conduct a TNA, the organizations need to define key concepts, including public health based on functions and services, public health workforce based on standard classification of occupations or job taxonomies relevant to their organizational environment, and the purpose of TNA. These pose many challenges including difficulties to define the people who work in public health,^5^^,^^6^ and who ‘hardly ever sit conveniently under the responsibility of any single organization’.^6^ The WHO-ASPHER Roadmap for Professionalising Public Health Workforce in the European Region divided the public health workforce into three categories: (i) Non–health sector professionals, (ii) Health and social care professionals and (iii) public health professionals.^6^ Public health workforce often does not acquire the necessary skills to perform their jobs successfully, which can be because some public health organizations or institutions do not carry out an adequate assessment of training needs due to a lack of proper definitions, lack of time or lack of knowledge about specific TNA tools and methodologies. These deficiencies have been documented in Africa,^7^ Europe,^8^ Latin America and the Caribbean.^9^

This review attempts to identify and describe TNA studies including tools and methodologies used in public health and health-related fields, with the aim of making them accessible to public health institutions and policymakers.

## Methods

A systematized review of the relevant literature related to tools used in training needs assessment for capacity building of the public health workforce was carried out in February 2022. Cochrane Handbook for systematic review version 5.2.0 and PRISMA 2020 statement were used to guide reporting. Original research, reports and grey literature from the websites of public health organizations in English were included. The search was conducted between 6 and 16 February 2022, and was centered around three key domains: (i) public health workforce and education, (ii) assessment and tools and (iii) training needs and capacity building (Supplementary data 2).

A focused search strategy was implemented using a set of key search terms. We used two databases (i) MEDLINE via PubMed to search for index literature and (ii) Google Scholar to conduct a search in grey and index literature. Additionally, a hand search was done on Google by using the search ‘training needs assessment in public health’ and searching the first 100 hints. Databases’ search contained keywords, indexed language and their synonyms, which were used in the title, abstract or as Mesh term. The retrieved literature was assessed for eligibility and no relevant results were excluded in phases: (i) title, (ii) full text and (iii) eligibility criteria, described per domain (Supplementary data 3). A consultation exercise was done among researchers and their networks to gather literature about TNA. Afterward, two researchers independently were involved in the screening process and found agreement on the final selection.

For the data collection, the extraction table was created, and it was piloted in five documents to determine its viability for this review. The data extracted were reported in the extraction table under the following headings: publication number (#), author/title/year of publication, country/location and organization, workforce studied, the objective of assessment (organizational/individual), tool’s name, methods used for the development of the tool, methods used for assessment and availability of the tool. Lastly, the data on the usefulness of TNA to improve training needs in practice was extracted by reading the conclusions, implications for policy and practice, and recommendations. Two researchers independently extracted the information from the different selected items, and differences were resolved by discussion of disagreements. The information was collected in verbatim quotes.

The information about the workforce studied was further categorized based on ‘employer type’, ‘what functions the worker performed’, ‘the worker’s occupation’, the ‘worker’s prior training’, ‘self-identify’^5^ (Supplementary data 1), and authors’ own definition. A document could have more than one category. Additionally, the objective of the assessment was categorized under three labels ‘training needs self-assessment’, ‘organizational assessment’ or ‘both’. To study the methods used for the assessment, the authors used previously identified methods such as surveys, focus groups, interviews or Delphi.^10^

The authors used Tableau Public 2021.4 to visualize the countries that conducted TNA worldwide. The tables were created using Microsoft Word. The quality and robustness of the documents reporting on TNA were examined by using the Joanna Briggs Institute’s (JBI) critical appraisal tools including cross-sectional and qualitative research, among others.^11^ JBI does not have a toolkit for mix-methods studies, therefore the mixed methods appraisal tool version 2018 was used^12^ (Supplementary data 4). The authors identified three documents, which reported on the tools that could be easily adapted to serve as TNA questionnaires and provide value for the designers of TNA. These documents did not undergo a quality check.

## Results

The search strategy identified 12 107 hits. After reading the titles, abstracts and full text applying inclusion criteria and quality assessment, 38 documents were included for evidence synthesis (Supplementary data 5 and 6). Thirty-five documents found that organizations have created their own tools to assess training needs in their public health workforce at institutional and individual levels (Supplementary data 7 and 8). Three documents identified organizations that created their own frameworks that could be adapted into a tool to assess TNA (Supplementary data 9).

Out of 38 studies, 27 documents were indexed literature (71%) and 11 grey documents (29%). TNA documents were published from 1999 to 2022, seven publications were published in 2019 (19%), five publications in 2020 (14%), five publications in 2015 (14%), four publications in 2021 (11%), four publications in 2018 (11%), two publications in 2022 and 2017 (6%) and one publication in 2014, 2012, 2010, 2007, 2005, 2001 and 1999. Two TNA did not have a publication year.

**Figure 1:**
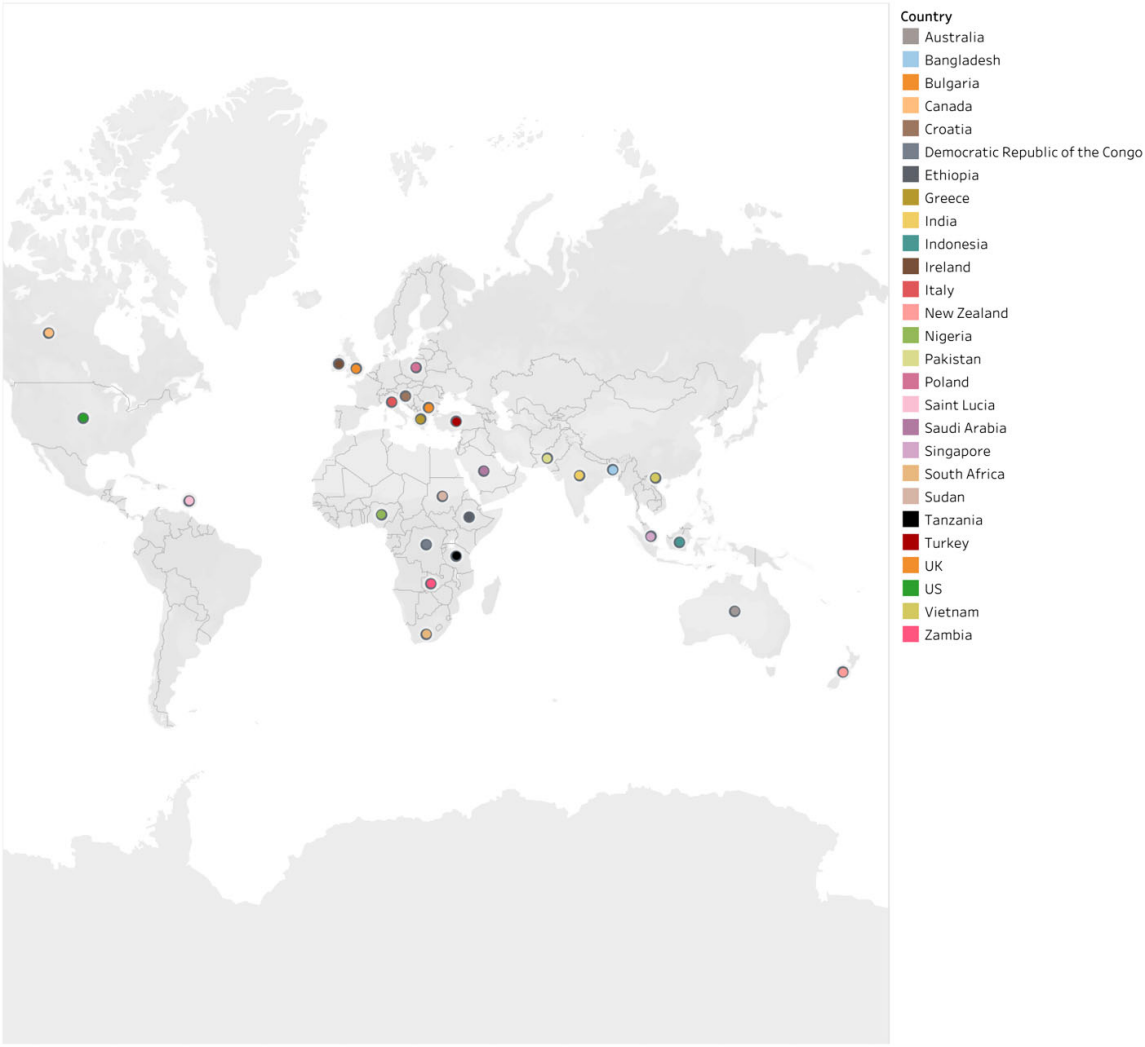
Countries that have conducted the TNA: training needs assessment tools for public health workforce at institutional and individual level—a review (*n* = 38)

TNA was performed in the following countries: Australia, Bangladesh, Canada, Croatia, the Democratic Republic of the Congo, Ethiopia, India, Indonesia, Nigeria, Pakistan, Saudi Arabia, the UK, the USA, Vietnam and Zambia (figure 1). One TNA was conducted in multiple countries such as Bangladesh, the Democratic Republic of the Congo, Ethiopia, India, Indonesia and Nigeria. One study presented a systematic review of 35 TNA using the Hennessy–Hicks questionnaire.^13^ This tool was used in Australia, Bulgaria, Greece, India, Indonesia, Italy, Ireland, New Zealand, Nigeria, Poland, Saint Lucia, South Africa, Singapore, Sudan, Tanzania, Turkey, the UK and the USA.

As categorization of the workforce, 19 papers used ‘employer type’ in their TNA. These included: regulatory authorities, research institutions, governmental institutions and ministries, insurance firms and other cooperating partners, NGOs, and universities (table 1). Five TNA studies were focused ‘on functions the workforce performed’ such as academic institutions’ staff, Synthesis and Translation of Research and Innovations from Polio Eradication (STRIPE), experts from the health, environment, agriculture, and rural development sectors at the provincial level or clinicians. The ‘self-identify’ category was not found in any of the selected publications.

**Table 1:** Workforce studied: training needs assessment tools for public health workforce at institutional and individual level—a review *(n *=* *38)

Workforce studied	Examples	Publication # as listed in Supplementary material 5
Employer type	Regional local governmental public health workforce US state and local governmental public health workers Local health departments Regulatory authorities, research institutions, government ministries, insurance firms and other cooperating partners State Health Agency-Central Office (SHA-CO) CDC full-time staff Bloomington Public Health staff and leadership Faculty members (The Medical Education Unit of the College of Medicine) NGO	1, 3, 4, 7, 10, 11, 12, 13, 14, 15, 16, 21, 22, 23, 25, 26, 30, 31, 33
Functions	Academic institutions’ staff. Synthesis and Translation of Research and Innovations from Polio Eradication’ (STRIPE) Experts from health, environment, agriculture and rural development sectors at provincial level Health workers offering immunization services Workforce capacity required to ensure effective delivery of essential public health service The workforce to deliver public health service	5, 8, 17, 18, 19
Occupation	Clinicians Epidemiologist Administration, Educator, Manager, Policy Officer, Researcher, and Technical Informatics; information technology (IT); clinical and laboratory; and other public health science specialists Public health emergency responders District managers Health manager	2, 9, 20, 23, 27, 28, 29, 32
Prior training	Doctor Nurse Midwife	6, 9, 36, 37, 38
Self-identify	None	None

Eight TNA reported ‘the workers' occupation’ such as epidemiologist, administrator, educator, manager, policy officer, researcher, technician, IT specialist, public health emergency responder, district manager or health manager. Five documents reported prior training, including nursing, medicine, and midwifery, and two documents provided their own definition of the occupation (table 1). Thirty-five documents stated that organizations developed their own tools to assess training needs in their public health workforce. The selected publications reported training needs self-assessment, organizational self-assessment or both. These were used in 26/35 studies (74%), 2/35 (7%) and 7/35 (20%).

Public Health Workforce Interests and Needs Survey (PH WINS)^14^ was the most common tool used for self-assessment in the USA and was used in 29 TNA. The research capacity and culture (RCC) tool was used in Australia. Markaki *et al*.^13^ discovered that the Hennessy–Hicks questionnaire was used in 18 countries, including Australia and the USA. Others created their own tools to assess organizational assessment and self-assessment (table 2). The TNA tools were created through the process of conducting expert panels, literature reviews, stakeholder interviews and quantitative surveys (table 3).

**Table 2:** Objective of assessment and its utility: training needs assessment tools for public health workforce at institutional and individual level—a review (*n *=* *35)^a^

Publication # as listed in Supplementary material 5	Objective of assessment	Information to improve training needs in practice
1	Organizational assessment	Local Health Departments (LHD) staff in Region V have a need for training related to the strategic skill domains of budgeting and financial management, change management, cultural competency and data for decision making. Continued assessment of training gaps is needed in order to understand if and how priority gaps have changed in light of the coronavirus pandemic.
2	Training needs self-assessment	Skill gaps decreased with increasing years of experience working in public health, suggesting that mentorship programs aimed at linking epidemiologists with less experience to those with more experience could be an effective strategy to leverage existing agency expertise to increase knowledge of new employees. State health agency central offices’ (SHA-CO) epidemiologists reported little engagement in some important epidemiologists’ self-reported awareness of emerging areas of public health practice (EAoP), including Health in All Policies, which may benefit from the epidemiologist’s skill set. Public health leaders should promote awareness of the skills epidemiologists offer to support EAoP to increase their engagement in these areas.
3	Training needs self-assessment	These results further illustrate the training gaps in financial planning and policy development. These results are useful for designing and implementing public health training systems and plans.
4	Training needs self-assessment	From a practice perspective, public health graduates have a strong foundation for work at the non-supervisor level. Therefore, recruiting graduates may be strategic, especially in terms of graduates from Bachelor of Public Health programs as these graduates may be more inclined to take entry-level positions. There were no significant differences for any of the competency skills within three particular domains: communication, budgeting and financing, and change management regardless of supervisory level. Public health agencies may need to address these gaps among their workers who are former graduates until the newly revised core competencies become more integrated into formal public health curricula and these graduates enter the workforce.
5	Both	The five factors that emerged from this research as relevant to readiness to conduct knowledge translation (KT) highlight unique contextual influencers and opportunities for capacity strengthening in low middle-income countries (LMICs). Five factors emerged as relevant for readiness to conduct KT in LMICs: institutional climate, organization change efficacy, prioritization and cosmopolitanism, self-efficacy, and financial resources. The organizational focus of these factors further points to a need for capacity building that includes but goes beyond individual training. Future research will be conducted to further understand the influencers of these readiness factors and systematically develop capacity building strategies for academic institutions in LMICs to conduct KT.
6	Both	The training needs assessment (TNA) instrument allows for triangulation of (i) assessment (identifying and triaging needs); (ii) needs (gap between what exists and what is required); and (iii) training (acquiring knowledge, skills or change attitude). TNA instrument has been widely used as a clinical practice and educational quality improvement tool across continents. Translation, cultural adaptation, and psychometric testing within a variety of settings, populations, and countries consistently reveal training gaps along the individual, team/interprofessional, and organizational themes. It is not only applied to identify training needs and demographic trends, but also to prioritize targeted training strategies and continuous professional development (CPD) programs. It facilitates triaging and allocating limited educational resources, especially in low and middle-income countries
7	Training needs self-assessment	The Region V Public Health Training Center (RVPHT) will first prioritize addressing training gaps that are shared across the six states. In particular, these will be the focus of self-paced trainings for skill development as well as interactive, peer-to-peer learning opportunities. Secondary priorities will be those top training gaps expressed by individual states and their subgroups. These will be explored primarily through training mechanisms such as webinars, podcasts, etc. Public health workforce development can be thought of as broadly including efforts related to ‘monitoring and projecting workforce supply, identifying competencies on which to base curricula, designing integrated learning systems, promoting public health practice competencies, conducting evaluations of and research on workforce development efforts, and ensuring support for lifelong learning’, with an emphasis on evidence-based practices that address the social determinants of health at the population level.
8	Both	Quantitative and qualitative results of this training need assessment showed that there was an urgent need in developing training programs to help building competencies in environmental health at master level for staff working in the health, environment and related sectors in Vietnam. For the next five years, the Master of Public Health majoring in environmental health, which is based on the current master of public health program, should be developed and implemented. In the longer term (e.g. in the next 5 to 10 years), the Master of Environmental Health program should be developed to meet the training needs in the country.
9	Training needs self-assessment	Undertaking this TNA survey has assisted the library’s training team to formulate strategies that address their clients’ information needs. It has been an important first step in obtaining evidence to guide the future of library services. The services the librarians provide will potentially increase research capacity, output and publishing that will support the department’s strategic direction and ultimately improve patientcare and outcomes
10	Both	This study assessed the skill gaps and mismatches in health policy and systems, health services management and planning, and health economics in the Zambian health sector. The study found significant skill gaps across all the three disciplines and significant skill mismatches were identified in health economics, and health services management and planning. We recommend a continuous assessment of public health training needs, given the ever-changing training needs of the health sector. Such reviews will help academics to tailor public health training to local context needs.
11	Training needs self-assessment	This study demonstrates the individual research capacity for medical, allied health and nursing professionals are different. Research capacity building needs to be individually tailored to the specific needs of each profession. This research will inform future capacity building activities and training for health professionals in a large public health organization of Sydney, Australia.
12	Training needs self-assessment	Skill gaps are prevalent and not merely among non-supervisors but also for supervisors, managers and executives. Agencies and support institutions, such as public health training centers, ought to tailor high-quality distance training to address these needs by the supervisory tie
13	Organizational assessment	Insights and opinions were collected from those who know the CDC responder training system the best: CDC responders themselves. From those perspectives, practical and actionable recommendations for the enhancement of CDC’s responder training system were developed and later translated to actionable recommendations that have been prioritized for implementation. Needs assessments of this scale should be conducted periodically as a building block for a continuously improving training system that is flexible and responsive to the evolving training needs of the responder workforce given the complexity of domestic and global health emergencies
14	Training needs self-assessment	Use the Public Health Workforce Interests and Needs Survey (PH WINS) training needs data to inspire coordination in the development of training that builds capacity in strategic skills in response to the needs identified.
15	Training needs self-assessment	The 2017 fielding of PH WINS provides a national benchmark for crosscutting training needs for the state and local governmental public health workforce. The largest areas of training need for the workforce are in budgeting and financial management, systems and strategic thinking, change management, and developing a vision of a health community
16	Training needs self-assessment	As new public health frameworks—like Public Health 3.0 and the Chief Health Strategist—are advanced nationally, it is necessary to ensure that the workforce has the skills and abilities to implement these frameworks. Those skills and abilities are precisely what are identified here. It is the responsibility of funders (federal and philanthropic), schools and programs of public health, national training centers, and state and local health department leaders to ensure that the training needs expressed by the workforce here and in previous studies are met. Otherwise, without a workforce with the necessary preparation to meet a changing environment, the health of the nation not only risks continued improvements, it risks decline.
17	Both	This TNA identified knowledge and skills gap among routine immunization service providers and tutors across major Expanded Program on Immunization thematic areas. The study also demonstrates that conducting TNAs is an important prerequisite for effective training because of the value of exposing not just knowledge gaps but other unmet training needs among service providers and facilitator.
18	Both	While a study such as PH WINS can identify broad needs in the field (e.g. communication, policy development or data analysis), Regional Public Health Training Centers (RPHTCs) are well suited to conduct more detailed assessments based on a subset of core questions (qualitative and quantitative) aligned with the identified needs. This approach would help clarify and prioritize needs and develop training to address these concerns.
19	Training needs self-assessment	Provide training opportunities in financial management, especially in budgets. Target training specific to certain areas in competencies with non-uniform levels of awareness and proficiency. Offer all employees … training in professional development and interpersonal skills. At the same time, offer leadership skills training to all employees, especially those with a supervisory role.
20	Training needs self-assessment	Sizable knowledge gaps exist in five of the nine main activities that state health agencies (SHA) epidemiologists rated as important in their daily work. The lessons learned from on-the-job training could be incorporated into public health continuing education programs, as nearly a quarter of epidemiologists reported no opportunities to receive on-site training from their jobs. Ongoing education and training are needed to maintain a competent and skilled applied epidemiology workforce.
21	Training needs self-assessment	Public health workers who self-report proficiency with business skills report increased job satisfaction, higher annual salaries and a more supportive training environment. These findings support the need for the development of appropriately designed business skill training opportunities to increase competencies in this critical domain at all levels of the public health workforce. Change is needed to create a core foundation of business skill knowledge that reaches a broader audience of the public health workforce.
22	Training needs self-assessment	It is essential to strengthen the communication between public health leaders and staff regarding the importance of workforce skills. While leaders should clearly convey their expectations and priorities, they should also actively seek and incorporate staff input into workforce development plans and programs
23	Training needs self-assessment	Results from PH WINS establish a baseline against which future growth and maturation of the public health informatics workforce, as well as expanding and evolving informatics training needs for the broader workforce, can be measured.
24	Training needs self-assessment	Investments in training for the existing public health workforce in policy analysis and development, business and financial management, systems thinking and social determinants of health, evidence-based public health practice, and collaborating with and engaging diverse communities. These topics are covered in the Core Competencies, which should be used to develop the curricula and evaluate the training.
25	Training needs self-assessment	Staff training plans will be developed based on the final result of the Core Competency High Yield analysis. As such, priorities for training will focus on those resources that will best develop higher priority areas where competency is relatively low and leverage higher priority areas where competency is relatively high. Development of financial planning and management skills. Leveraging of cultural competency, leadership and systems thinking and communication skills.
26	Training needs self-assessment	Through our needs assessment survey, we demonstrated a participatory approach which is simple, feasible and useful in the Gulf Region context and should also be in other regions. We focused on identifying the gap between faculty’s perceived importance and self-rated performance in 12 skill areas, as criteria for prioritizing faculty development content.
27	Training needs self-assessment	New competencies based on local needs were identified that provide coverage of subject matter appropriate to local public health emergency responders beyond the focus of existing national competency sets.
28	Training needs self-assessment	This article describes … in the development of a tool to assess agencies’ training readiness using five factors derived from learning organization theory. These factors (resources, policies, learning culture, programs and leadership) offer a useful framework for further development of a tool to assess training program readiness.
29	Both	The Punjab TNA set out to tackle problems identify by the Second Family Health Project: lack of clarity on the roles between different levels of the services; duplication of functions; decision-making structures without community representation; poor supervision; low motivation of personnel; and poor management of information and finances. The TNA identified where current training was failing to address these problems, and where the felt needs of the health staff coincided with organizational needs.
30	Training needs self-assessment	Note: the refence just provides the tool.
31	Training needs self-assessment	Note: the refence just provides the tool.
32	Training needs self-assessment	Note: the refence just provides the tool.
33	Training needs self-assessment	Note: the refence just provides the tool.
34	Training needs self-assessment	Note: the refence just provides the tool.
35	Training needs self-assessment	Note: the refence just provides the tool.
Summary	Training needs self-assessment = 26 Organizational assessment = 2 Both = 7	To conduct TNA to enhance KT in LMICs. To explore the coherence between the formal public health curricular programs and the vital competencies to work in public health organizations. To prepare the public health workforce for pandemics such as COVID-19. To call for mentorship programs to reduce competency gaps in the public health workforce with less experience. To reveal the training gaps in three themes: individual, team/interprofessional and organizational. To prioritize targeted training strategies and CPD. To design and implement public health training systems and plans within organizations. To improve public health services To reduce organizations’ challenges such as: lack of clarity on the roles between different levels of the services; duplication of functions; decision-making structures without community representation; poor supervision; low motivation of personnel; and poor management of information and finances.

a 36, 37 and 38 are competency frameworks that one can adapt to become a tool.

**Table 3:** Methods used for the assessment: training needs assessment tools for public health workforce at institutional and individual level—a review *(n *=* *35)^a^

Publication # as listed in supplementary material 5	Survey (X)	Interviews (X)	Focus group (X)	Delphi techniques (X)	Questionnaires (X)	Matrix (X)	Total tools
1	X						1
2	X						1
3	X						1
4	X						1
5	X						1
6					X		1
7	X						1
8	X	X	X	X	X		5
9	X						1
10					X		1
11	X						1
12	X						1
13		X	X				2
14	X						1
15	X						1
16	X						1
17	X		X				2
18	X						1
19	X	X			X		3
20	X						1
21	X						1
22	X						1
23	X						1
24	X						1
25					X		1
26	X						1
27						X	1
28					X		1
29		X	X				2
30	X						1
31					X		1
32					X		1
33	X						1
34	X						1
35	X						1
Total	26	4	4	1	8	1	

a 36, 37 and 38 are competency frameworks that one can adapt to be a tool.

Organizations created 35 tools to conduct TNA in their public health workforce. The TNA tools took the form of a survey (26/35, 74%), questionnaire (8/35, 22%), interviews (4/35, 11%), focus groups (4/35, 11%), Delphi (1/35, 2%) and matrix (1/35, 2%). Some studies used multiple tools to carry out the assessment. Two competency frameworks were identified as suitable to assess TNA by nursing organizations: (i) the American Nurses Association or (ii) the American Organisation of Nurse Executives developed competency frameworks. Three tools were identified in this review as relevant examples to assess the competency levels of individuals. These are (i) the First Nations Health Manager Competency Framework Self-Assessment Tool, (ii) Forces of Change Survey (FoC) 2018 and (iii) Public Health Skills and Knowledge Framework.

These tools to conduct TNA provided useful information to improve training needs in practice (table 2). For instance, they can help enhance knowledge translation (KT) in low middle-income countries (LMIC), prepare the public health workforce (PHW) for pandemics such as COVID-19, disclose training gaps at individual, team and organizational levels, explore the coherence between the formal public health curricular programs and the vital competencies needed to work in health organizations, call for mentorship programs to reduce competency gaps in the public health workforce with less experience, prioritize targeted training strategies and continuing professional development (CPD), or reduce organizations’ challenges such as: lack of clarity on the roles at different levels of the services, duplication of functions, existence of decision-making structures without community representation, poor supervision, low motivation of personnel, and poor management of information and finances.

## Discussion

This review provides evidence that many organizations have been using the TNA of the public health workforce to find and evaluate training gaps and assess capacities that are essential to delivering organizations’ functions and services. Some of the identified TNA tools can provide guidance when performing a TNA of the public health workforce. For instance, (i) the Hennessy–Hicks questionnaire, (ii) PH WINS 2014 and 2017, (iii) Forces of Change (FoC) 2015, (iv) the Public Health Skills and Knowledge Framework (PHSKF) 2016 and (v) the Centers of Disease Control and Prevention’s (CDC) tool 2019. However, there are multiple challenges when performing a TNA: (i) the definition of the study population, (ii) the type of assessment (individual, organizational or both) and (iii) methods and tools to collect data.

To conduct a TNA, the organizations need to define key concepts such as a definition of public health based on functions and services, and a definition of public health workforce based on standard classification of occupations or job taxonomies relevant to their organizational environment. These pose many challenges including difficulties to define the people who work in public health and with specific functions within the organization.

Watts *et al*.^5^ in their systematic review provided four definitions of the public health workforce that are also used in this study. PH WINS survey, which is directed toward the American public health workforce, has a very robust demographics section that includes among other questions such as ‘Identify the classification that best represents your role in the organization’ and ‘indicate which degrees you have attained’. These questions are in line with Watts *et al*.’s^5^ recommendations to describe the functions performed and the education of the workforce replying to the assessment. However, PH WINS does not use international standard classifications of demographic characteristics, so the results cannot be compared across countries. The Hennessy–Hicks questionnaire which is more directed towards a clinical environment than PH WINS gathers information about the public health workforce by asking about individuals’ job titles without collecting data on the functions performed and education.^16^^,^^17^ On the other hand, CoP, CDC’s tool, and PHSKF do not collect information about the public health workforce’s demography. Consequently, each tool does not allow for comparing and contrasting public health workforce’s definitions across countries. In fact, Watts et al. recommend reporting the public health workforce’s occupations, which should be aligned with the international standard classification of occupations (ISCO), such as ISCO-08.^5^^,^^18^

The second challenge is to choose at what level, organizational, individual or both, the TNA should be conducted. The results of this study show that the training needs self-assessment at the individual level is the most frequent assessment. The European Centre for Disease Prevention and Control (ECDC) conducted a review to propose a methodology for assessing training needs in the European Union (EU) Member States and European Economic Area (EEA) countries,^10^ which guided the organization of triennial surveys.^8^^,^^19^^,^^20^ In addition, in the area of infection control and hospital hygiene (IC/HH), three surveys were conducted in 2006, 2010 and on workforce capacity and training needs.^21^

ECDC assessments were more oriented to organizational TNA rather than individual ones,^8^,^19–21^ and this study fills this gap by exploring the best practices and examples of both individual and organizational assessments. For instance, public health organizations used PH WINS for training needs self-assessment,^22–25^ Hennessy–Hick’s questionnaire was used for training needs self-assessment and organizational assessment,^13^ CoP for training needs self-assessment,^25^ and CDC’s tool for organizational assessment^15^ as documented in this study.

The third challenge is determining which methods and techniques the organizations use or will use for TNA. Consistent with the ECDC, which has used questionnaires, surveys, and literature review to conduct TNA since 2015,^8^,^19–21^ this review shows that there is a variety of methods to conduct TNA. Therefore, public health organizations and the public health workforce can apply a combined methodology for TNA in line with the ECDC’s claim that a combined methodology is preferable to create an effective tool for TNA.^20^ Organizations have used surveys, interviews, focus groups, Delphi techniques, questionnaires and a matrix. These are usually based on competency frameworks or competency statements, which reflect the main knowledge and skills needed to perform essential functions in the organization.^10^ These methods can provide qualitative and quantitative data. The PH WINS survey, FoC and PHSKF gather mainly quantitative information while the CDC gathers also qualitative data.^15^

It is also of note that some TNA assessments might seem to assume a pragmatic approach from the start in anticipation of how the results of the assessment may change the programs. For example, O’ Meara *et al*.^15^ identified the perceived preparedness and response training needs for the CDC personnel. They conducted focus groups and interviews using the following questions: ‘How well does the current training system prepare CDC staff to respond to emergency events?’, ‘What gaps exist in the current training system?’, and ‘What other existing or potential training is essential and should be included in the training system?’ The authors were able to identify three areas to enhance CDC performance by including training leaders working at the command center for incident management system (IMS) activations, and those who participated in the interviews and the focus group. In the first place, (i) potential IMS command staff should gain experience via shadowing or observing other command staff; (ii) coordinate IMS meetings related to domestic and international responses to share experiences and lessons learned and (iii) develop a formal career track for potential IMS staff with experiential training opportunities. Similarly, the CDC’s Office of Public Health Preparedness and Response, Division of Emergency Operations (DEO) accepted some recommendations from O’ Meara et al. research. For instance, DEO launched the role-specific Incident Management Training and Development Program to teach and train public health leadership capacity and integrate response across CDC programs. The curriculum for this leadership training was modular, designed as an infectious disease case study with a didactic component and included activities with CDC-specific examples, and strategic concepts woven throughout the module. All training was delivered by experienced CDC response leaders in a classroom environment with small groups to foster a team-based approach.

Finally, the organizations which want to do TNA can use known and published competency frameworks such as, e.g. ECDC Core Competencies in Applied Infectious Disease Epidemiology in Europe^26^ or WHO-ASPHER Competency Framework for Public Health workforce in the European Region,^27^ or any other competency framework, which reflects the competencies or skills that are needed in the organization.^28–30^ Some organizations can have their own competency lists or statements, which they use to describe roles or jobs.

This review focuses on the use of TNA in the area of the public health workforce; however, TNA can be successfully used in other health care settings including clinical care.^31^^,^^32^ Using TNA in the health system demonstrates its importance for organizations that deliver health care and public health services. Elmaraghi *et al*.^31^ aimed at identifying priorities of surgeon educators in African countries, and they used a survey to identify these priorities. They concluded that the most important domain was faculty development to assure correct infrastructure to conduct research for health (73% of the participants). Our review is in line with the findings of Elmaraghi et al. confirming that surveys are the most common method (26/35, 74% of publications) to conduct TNA. TNAs are also useful to provide recommendations to improve KT in a health care field in LMIC. KT is a process to conduct, implement, and assess research evidence to improve health outcomes, and KT aims at using the best possible resources such as workforce, money and time,^33^ therefore using TNA for KT is vital in LMIC, which have less resources than high income countries.

Lanza *et al*.^32^ conducted a systematic review of health care workforce training needs in vaccination and vaccine uptake in Europe. Our review and Lanza et al. review complement each other to study training needs. Lanza and colleagues structured their search strategy for training needs around the following terms: ‘knowledge, competency, attitudes, skill, practice, clinical competence and learning need’. On the other hand, this review uses two search strings to search TNA. The first one looks at ways to study training assessment such as ‘assess*, feedback, questionnaire, evaluat*, survey, tool and rubric’, and the second string is about needs including ‘training needs, competency needs, educational needs, skill needs, knowledge needs, learning needs, capacity building, staff development and capacity building’. Lanza et al. did not aim at studying tools for conducting TNA in a global context, they concluded that the health care workforce needs general knowledge of vaccine-preventable diseases in Europe. This review identified tools from multiple continents that individuals and organizations can use to conduct TNA in their organizational context. For instance, some European organizations which work on vaccination can use the US CDC’s TNA tool.

### Limitations

Although this study is direction-setting, there are some limitations. This study is not a systematic review of literature, which makes it slightly less rigorous concerning the identified and selected sources. However, the nature and aim of this study justify the choice of the method. Nevertheless, the authors applied the necessary quality checks and appraisal tools to select the articles for the final review.

## Conclusion

TNA is useful for defining and characterizing public health workforce in every organization. It can also be used in other settings including clinical care. The workforces share organization’s mission, vision and objectives; however, they consist of individuals who have their own training needs to fulfil their tasks. Therefore, individual and organizational TNA using various assessment methods and tools should be combined to study the public health workforce and their capacities.

## Supplementary Material

ckad183_Supplementary_Data

## Data Availability

The data underlying this article are available in the article and in its online supplementary data.

## Disclaimer

This peer-reviewed article has been written based on the report ‘Public health workforce training needs assessment tools at institutional and individual level (2022)’, for which the European Centre for Disease Prevention and Control (ECDC) holds the copyright.

*Conflicts of interest*: None declared.
Key points

- Training needs assessment (TNA) is a useful tool to assess and understand public health workforce and their capacities that are essential to delivering organizations’ functions and services.
- TNA also identifies the relations between these levels, gaps in capacities, competencies and know-how.
- To conduct a TNA, the organizations need to define key concepts such as a definition of public health based on functions and services, and a definition of public health workforce based on standard classification of occupations or job taxonomies relevant to their organizational environment.
- Public health organizations use various methods to conduct a TNA including questionnaires, surveys, interviews, focus groups, Delphi techniques, questionnaires, a matrix, literature review or a combined methodology.
- There are multiple challenges when performing a TNA: (i) the definition of the study population, (ii) the type of assessment (individual, organizational or both) and (iii) methods and tools used to collect data.
