# Supplementary material for: Training needs assessment tools for the public health workforce at an institutional and individual level: a review
Source: Eur J Public Health. 2023 Oct 25;34(1):59–68. doi: 10.1093/eurpub/ckad183 (PMC10843943; doi:10.1093/eurpub/ckad183)
Supplement: ckad183_Supplementary_Data [file ckad183_supplementary_data.docx]

**Supplementary material**

**Training needs assessment tools for the public health workforce at an institutional and individual level – a review**

| Supplementary material 1 Glossary of Terms: Training needs assessment tools for public health workforce at institutional and individual level - a review. | | |
| --- | --- | --- |
| Key word | **Definition** | **Reference** |
| Capacity Assessment | A capacity assessment identifies capacity on three levels: individual, organizational and enabling environment. It is a more comprehensive approach than training needs assessment as it also looks at the interrelation between each of these levels of capacity | United Nations Human Settlements Programme 2012. Manual Training Needs Assessment and Training Outcome Evaluation In an Urban Context. 2012. Available from: Training Needs Assessment and Training Outcome Evaluation, In and Urban Context \| UN-Habitat (unhabitat.org)Accessed on 9^th^ July 2023 |
| Capacity Development | The process whereby people, organizations and society as a whole unleash, strengthen, create, adapt, and maintain capacity over time | United Nations Human Settlements Programme 2012. Manual Training Needs Assessment and Training Outcome Evaluation In an Urban Context. 2012. Available from: Training Needs Assessment and Training Outcome Evaluation, In and Urban Context \| UN-Habitat (unhabitat.org)Accessed on 9^th^ July 2023 |
| Competencies | Actions that require multiple skills, knowledge, and particular attitudes or values | Rodríguez-Feria P, Flórez LJH, Czabanowska K. Leadership Competencies for Knowledge Translation in Public Health: A consensus study. J Public Health (Oxf). 2021 |
| employer type | (e.g., employees of a local health center and members of a college of physicians) | Watts RD, Bowles DC, Ryan E, Fisher C, Li IW. No Two Workforces Are the Same: A Systematic Review of Enumerations and Definitions of Public Health Workforces. Front Public Health. 2020;8:588092 |
| Evaluation | Determining the size of a professional competence gap and the impact it may bring on activities | United Nations Development Programme Moldova. On Training Needs Assessment of the National Institutions for the Human Rights Protection and Promotion. 2015. Available from:procurement-notices.undp.org.doc (live.com) Accessed on 9^th^ July 2023 |
|  |  | (Continued) |
| Supplementary material 1 Continued |  |  |
| Key word | **Definition** | **Reference** |
| **Individual assessment** | “Analyses how well an individual employee is doing a job and determines the individual's capacity to do new or different work. Individual assessment provides information on which employees need training and what kind” | U.S Office of Personnel Management. Policy, Data, Oversight TRAINING AND DEVELOPMENT: Training Needs Assessment. Available from: Planning & Evaluating (opm.gov) Accessed on 9^th^ July 2023 |
| Institutional capacities | Internal management systems, policies and procedures of an organisation, including professional capacities/skills of the employees | United Nations Development Programme Moldova. On Training Needs Assessment of the National Institutions for the Human Rights Protection and Promotion. 2015. Available from:procurement-notices.undp.org.doc (live.com) Accessed on 9^th^ July 2023 |
| Institutional capacity assessment | Assessing the internal environment to establish the difference between the existing and required capacities to register the most optimal organisational/institutional performance | United Nations Development Programme Moldova. On Training Needs Assessment of the National Institutions for the Human Rights Protection and Promotion. 2015. Available from:procurement-notices.undp.org.doc (live.com) Accessed on 9^th^ July 2023 |
| Organizational assessment | “Evaluates the level of organizational performance. An assessment of this type will determine what skills, knowledge, and abilities an agency needs. It determines what is required to alleviate the problems and weaknesses of the agency as well as to enhance strengths and competencies, especially for Mission Critical Occupation's (MCO). Organizational assessment takes into consideration various additional factors, including changing demographics, political trends, technology, and the economy” | U.S Office of Personnel Management. Policy, Data, Oversight TRAINING AND DEVELOPMENT: Training Needs Assessment. Available from: Planning & Evaluating (opm.gov) Accessed on 9^th^ July 2023 |
|  |  | (Continued) |
| Supplementary material 1 Continued |  |  |
| Key word | **Definition** | **Reference** |
| **Occupational assessment** | “Examines the skills, knowledge, and abilities required for affected occupational groups. Occupational assessment identifies how and which occupational discrepancies or gaps exist, potentially introduced by the new direction of an agency. It also examines new ways to do work that can eliminate the discrepancies or gaps” | U.S Office of Personnel Management. Policy, Data, Oversight TRAINING AND DEVELOPMENT: Training Needs Assessment. Available from: Planning & Evaluating (opm.gov) Accessed on 9^th^ July 2023 |
| Public health | Public health is defined as the science of protecting the safety and improving the health of communities through education, policy making and research for disease and injury prevention. (CDC Foundation) | Public Health National Center for Innovations. THE 10 ESSENTIAL PUBLIC HEALTH SERVICES.2020. Available from: EPHS-English.pdf (phnci.org) Accessed on 9^th^ July 2023 |
| Public health | The art and science of preventing disease, prolonging life and promoting health through the organized efforts of society | World Health Organisation Regional Officer for Europe. Public Health Services. Available from: WHO/Europe \| Public health services Accessed on 9^th^ July 2023 |
| Public health | Public health is by definition everybody’s business, requiring multiple, multidisciplinary interventions and requiring the full engagement of the public | Roadmap to professionalizing the public health workforce in the European Region. Copenhagen: WHO Regional Office for Europe; 2022. Licence: CC BY-NC-SA 3.0 IGO. Available from: WHO-EURO-2022-4808-44517-63144-eng.pdf Accessed on 9^th^ July 2023 |
| Public health workforce | “Workforces varied in scope, from highly specific occupations (e.g., food safety epidemiologists) to disparate groups of people who provided a key function of public health e.g., anyone with the opportunity or ability to positively impact health through their work | Watts RD, Bowles DC, Ryan E, Fisher C, Li IW. No Two Workforces Are the Same: A Systematic Review of Enumerations and Definitions of Public Health Workforces. Front Public Health. 2020;8:588092 |
|  |  | (Continued) |
|  |  |  |
|  |  |  |
| Supplementary material 1 Continued |  |  |
| Key word | **Definition** | **Reference** |
| Public health workforce | “Almost all definitions were pragmatic in nature, with conceptual definitions commonly included as a survey item to help workers self-identify as part of the public health workforce” | Watts RD, Bowles DC, Ryan E, Fisher C, Li IW. No Two Workforces Are the Same: A Systematic Review of Enumerations and Definitions of Public Health Workforces. Front Public Health. 2020;8:588092 |
| Public health workforce | “Pragmatic definitions could generally be considered as defining a workforce on the basis of: employer type (e.g., employees of a local health centre and members of a college of physicians); what functions the workers performed (e.g., all workers who perform environmental health functions); the workers’ occupations (e.g., all epidemiologists and all persons who identified as part of the public health nutrition workforce); or the workers’ prior training (e.g., all persons with a Public Health degree)” | Watts RD, Bowles DC, Ryan E, Fisher C, Li IW. No Two Workforces Are the Same: A Systematic Review of Enumerations and Definitions of Public Health Workforces. Front Public Health. 2020;8:588092 |
| Public health workforce | When conceptual definitions were used, no two were identical but commonly focused mainly on the core ideas “population” and “health” [e.g., “anyone who works with groups and/or communities to protect, promote, or advance health/wellness.” | Watts RD, Bowles DC, Ryan E, Fisher C, Li IW. No Two Workforces Are the Same: A Systematic Review of Enumerations and Definitions of Public Health Workforces. Front Public Health.2020;8:588092 |
| Public health workforce | Those who influence the entire public health system by cultivating and curating the necessary inputs and processes through which population outcomes are achieved | Sellers K, Leider JP, Harper E, Castrucci BC, Bharthapudi K, Liss-Levinson R, et al. The Public Health Workforce Interests and Needs Survey: The First National Survey of State Health Agency Employees. J Public Health Manag Pract. 2015;21 Suppl 6:S13-27 |
|  |  | (Continued) |
|  |  |  |
|  |  |  |
| Supplementary material 1 Continued |  |  |
| Key word | **Definition** | **Reference** |
| Public health workforce | U.S. Department of Health and Human Services has defined public health workers as “all those responsible for providing the essential services of public health regardless of the organization in which they work  *U.S. Department of Health and Human Services  Gebbie, Merrill, and Tilson emphasize that a public health worker can be defined on different three dimensions: the specific profession (e.g., epidemiologist), the work setting (e.g., all local health department workers, regardless of profession), or the work or job function | University of Michigan/Center of Excellence in Public Health Workforce Studies, University of Kentucky/Center of Excellence in Public Health Workforce Research and Policy. Strategies for Enumerating the U.S. Governmental Public Health Workforce. Rev. ed. Washington, DC: Public Health Foundation; 2012. Available at: http://www.phf.org Accessed on 9^th^ July 2023 |
| self-identify | Almost all definitions were pragmatic in nature, with conceptual definitions commonly included as a survey item to help workers self-identify as part of the public health workforce | Watts RD, Bowles DC, Ryan E, Fisher C, Li IW. No Two Workforces Are the Same: A Systematic Review of Enumerations and Definitions of Public Health Workforces. Front Public Health. 2020;8:588092 |
| The workers' occupations | (e.g., all epidemiologists and all persons who identified as part of the public health nutrition workforce) | Watts RD, Bowles DC, Ryan E, Fisher C, Li IW. No Two Workforces Are the Same: A Systematic Review of Enumerations and Definitions of Public Health Workforces. Front Public Health. 2020;8:588092 |
|  |  | (Continued) |
|  |  |  |
| Supplementary material 1 Continued |  |  |
| Key word | **Definition** | **Reference** |
| The workers' prior training | (e.g., all persons with a Public Health degree) | Watts RD, Bowles DC, Ryan E, Fisher C, Li IW. No Two Workforces Are the Same: A Systematic Review of Enumerations and Definitions of Public Health Workforces. Front Public Health. 2020;8:588092 |
| Training Needs Assessment | A training needs assessment looks specifically at the skills, knowledge and attitudes of potential trainees. It uses this information to determine if and how the issue can be improved by training. | United Nations Human Settlements Programme 2012. Manual Training Needs Assessment and Training Outcome Evaluation In an Urban Context. 2012. Available from: Training Needs Assessment and Training Outcome Evaluation , In and Urban Context \| UN-Habitat (unhabitat.org) |
| Training Outcome Evaluation | A training outcome evaluation is carried out to see if the objectives of the training have been met and how the training process can be improved. Results can be measured on four levels: the event and the participants’ immediate reactions, the participants’ learning, the participants’ job performance, and the organizational performance | United Nations Human Settlements Programme 2012. Manual Training Needs Assessment and Training Outcome Evaluation in an Urban Context. 2012. Available from: 11406_1_594526.pdf (unhabitat.org) Accessed on 9^th^ July 2023 |
| what functions the worker performed | (e.g., all workers who perform environmental health functions) | Watts RD, Bowles DC, Ryan E, Fisher C, Li IW. No Two Workforces Are the Same: A Systematic Review of Enumerations and Definitions of Public Health Workforces. Front Public Health. 2020;8:588092. |

| **Supplementary material 2** Search Strategy**:** Training needs assessment tools for public health workforce at institutional and individual level - a review | | |
| --- | --- | --- |
| **PubMed Strategy: Database** | **Search strategy** | **Hits** |
| February 6^th^ 2022 | 1. Public health[Title/Abstract] 2. Public health workforce[Title/Abstract] 3. Education[Title/Abstract] 4. Education, Public Health Professional[MeSH Terms] 5. #1 OR #2 OR #3 OR #4 6. Assess*[Title/Abstract] 7. Feedback[Title/Abstract] 8. Questionnaire*[Title/Abstract 9. Evaluat*[Title/Abstract] 10. Survey*[Title/Abstract] 11. Tool*[Title/Abstract] 12. Rubric*[Title/Abstract] 13. #6 OR #7 OR #8 OR #9 OR #10 OR #11 OR#12 14. Training needs[Title/Abstract] 15. Competenc* needs [Title/Abstract] 16. educational needs [Title/Abstract] 17. Skill* needs[Title/Abstract] 18. knowledge needs[Title/Abstract] 19. learning needs[Title/Abstract 20. capacity building[Title/Abstract] 21. Staff Development[MeSH Terms] 22. Capacity building[MeSH Terms] 23. #14 OR #15 OR #16 OR #17 OR #18 OR #19 OR #20 OR #21 OR #22 24. # 5 AND # 13 AND #23 | 11 425 |

(Continued)

|  |  |  |
| --- | --- | --- |
| **Supplementary material 2** Continued | | |
| **Google Scholar: Database** | **Search strategy** | **Hits** |
| February 6^th^ 2022 | allintitle:" needs assessment" OR "training needs" OR "Competencies needs" OR " educational needs" OR "skills needs" OR " knowledge needs" OR "learning needs" OR "capacity building" OR "Staff Development " OR "Capacity building“ "public health“  English Language | 569 |
| **Google Scholar: Handsearching** | **Search strategy** | **Hits** |
| February 16^th^ 2022 | “Training needs assessment in public health” | 100 |

| **Supplementary material 3** Eligibility Criteria: Training needs assessment tools for public health workforce at institutional and individual level - a review. | | |
| --- | --- | --- |
| **Domain** | **Inclusion Criteria** | **Exclusion criteria** |
| Public health and workforce and education | 1. Public health workforce  2. Epidemiologist  3. Medicine (residency and fellows)  4. Nurses | 1. Undergraduate Medical Education.  2. Dentistry  3. Midwifery |
| Assessment and tools | 1. Training Needs Assessment (at individual and organisational level)  2. Self-assessment  3. Assessment by others | 1.Health Needs Assessment  2.Community Health Needs Assessments  3. Health Technology Assessment |
| Training needs and capacity building | 1. Training needs tools, research on training and/or capacity building needs assessment and/or capacity assessment in public health settings  2. Contain a description of a needs assessment methodology performed at a territorial/country or institutional level | Do not provide information on:  1. to assess the training needs of professionals (for Continuing Professional Development)  2. kind of tools used  3. kind of questions asked |
| Critical appraisal tools | The ≥80% of the checklist items were marked as   1. “yes” 2. “Non applicable” 3. “unclear” 4. “Cannot tell” | The ≥20% of the checklist items were marked as   1. “no” |

| Supplementary material 4 Quality appraisal: Training needs assessment tools for public health workforce at institutional and individual level - a review. | | |
| --- | --- | --- |
| # | **Author, Tittle, Year of Publication** | **Critical appraisal ( Tool and its considerations)** |
| 1 | Kulik PKG, Leider JP, Beck AJ. Leadership Perspectives on Local Health Department Workforce Development: A Regional Training Needs Assessment. J Public Health Manag Pract. 2022;28(2):E619-E23 | Checklist for Analytical Cross-Sectional Study:   1. Were the criteria for inclusion in the sample clearly defined? Not applicable (Inclusion criteria provided, exclusion not provided) 2. Were the study subjects and the setting described in detail? Yes. 3. Was the exposure measured in a valid and reliable way? Yes 4. Were objective, standard criteria used for measurement of the condition? Yes 5. Were confounding factors identified? Not applicable 6. Were strategies to deal with confounding factors stated? Not applicable 7. Were the outcomes measured in a valid and reliable way? Yes 8. Was appropriate statistical analysis used? Yes   Overall appraisal: Include |
| 2 | Daly ER, Gendelman M, Arrazola J, Sellers K. Training Needs and Awareness of Emerging Areas of Public Health Practice Among Epidemiologists Working in State Health Agency Central Offices in the United States, 2017. J Public Health Manag Pract. 2022;28(1):E273-E82. | Checklist for Analytical Cross-Sectional Study:   1. Were the criteria for inclusion in the sample clearly defined? Not applicable (Inclusion criteria provided, exclusion not provided) 2. Were the study subjects and the setting described in detail? Yes 3. Was the exposure measured in a valid and reliable way? Not applicable 4. Were objective, standard criteria used for measurement of the condition? Yes 5. Were confounding factors identified? Not applicable 6. Were strategies to deal with confounding factors stated? Not applicable 7. Were the outcomes measured in a valid and reliable way? Yes 8. Was appropriate statistical analysis used? Yes   Overall appraisal: Include |
| 3 | Sainkhuu S, Cunha-Cruz J, Rogers M, Knerr S, Bekemeier B. Evaluation of Training Gaps Among Public Health Practitioners in Washington State. J Public Health Manag Pract. 2021;27(5):473-83. | Checklist for Analytical Cross-Sectional Study:   1. Were the criteria for inclusion in the sample clearly defined? Not applicable (Inclusion criteria provided, exclusion not provided) 2. Were the study subjects and the setting described in detail? Yes 3. Was the exposure measured in a valid and reliable way? Not applicable 4. Were objective, standard criteria used for measurement of the condition? Yes 5. Were confounding factors identified? Not applicable |
|  |  | (Continued) |
| Supplementary material 4 Continued | | |
| # | **Author, Tittle, Year of Publication** | **Critical appraisal ( Tool and its considerations)** |
|  |  | 1. Were strategies to deal with confounding factors stated? Not applicable 2. Were the outcomes measured in a valid and reliable way? Yes 3. Was appropriate statistical analysis used? Yes.   Overall appraisal: Include |
| 4 | Taylor HL, Yeager VA. Core Competency Gaps Among Governmental Public Health Employees With and Without a Formal Public Health Degree. J Public Health Manag Pract. 2021;27(1):20-9. | Checklist for Analytical Cross-Sectional Study   1. Were the criteria for inclusion in the sample clearly defined? Yes (inclusion and exclusion) 2. Were the study subjects and the setting described in detail? Yes 3. Was the exposure measured in a valid and reliable way? Yes 4. Were objective, standard criteria used for measurement of the condition? Yes 5. Were confounding factors identified? Not applicable 6. Were strategies to deal with confounding factors stated? Not applicable 7. Were the outcomes measured in a valid and reliable way? Yes 8. Was appropriate statistical analysis used? Yes   Overall appraisal: Include |
| 5 | Kalbarczyk A, Rao A, Alonge O. A mixed methods study to develop a tool to assess institutional readiness to conduct knowledge translation activities in low-income and middle-income countries. BMJ Open. 2021;11(10):e050049. | Mixed Methods Appraisal Tool (MMAT), version 2018  Screening questions (for all types)  S1. Are there clear research questions? Yes  S2. Do the collected data allow to address the research questions? Yes  1-4 Quantitative randomized controlled trials, Quantitative randomized controlled trials and Quantitative randomized controlled trials: Comments Not applicable. Authors used a literature review and stakeholder consultation ( interviews and surveys)  5. Mixed methods  5.1. Is there an adequate rationale for using a mixed methods design to address the research question? Yes  5.2. Are the different components of the study effectively integrated to answer the research question? Yes  5.3 .3. Are the outputs of the integration of qualitative and quantitative components adequately interpreted? Yes |
|  |  | (Continued) |
|  |  |  |
| Supplementary material 4 Continued | | |
| # | **Author, Tittle, Year of Publication** | **Critical appraisal ( Tool and its considerations)** |
|  |  | 5.4. Are divergences and inconsistencies between quantitative and qualitative results adequately addressed? Yes  5.5. Do the different components of the study adhere to the quality criteria of each tradition of the methods involved? Yes  Overall appraisal: Include |
| 6 | Markaki A, Malhotra S, Billings R, Theus L. Training needs assessment: tool utilization and global impact. BMC Med Educ. 2021;21(1):310. | Toolkit: checklist for systematic reviews and research syntheses   1. Is the review question clearly and explicitly stated? Yes 2. Were the inclusion criteria appropriate for the review question? Yes 3. Was the search strategy appropriate? Yes 4. Were the sources and resources used to search for studies adequate? Yes 5. Were the criteria for appraising studies appropriate? Yes 6. Was critical appraisal conducted by two or more reviewers independently? Yes. 7. Were there methods to minimize errors in data extraction? Yes 8. Were the methods used to combine studies appropriate? Unclear 9. Was the likelihood of publication bias assessed? Unclear (out of the scope in this review) 10. Were recommendations for policy and/or practice supported by the reported data? Yes 11. Were the specific directives for new research appropriate? Yes   Overall appraisal: Include |
| 7 | Region V Public Health Training Center. 2020 Training Needs Assessment Survey.2020. Available from Training Needs Assessment_2020_RVPHTC.pdf - Google Drive Accessed on 24 ^h^  March 2022 | Toolkit: Qualitative research   1. Is there congruity between the stated philosophical perspective and the research methodology? Yes 2. Is there congruity between the research methodology and the research question or objectives? Yes 3. Is there congruity between the research methodology and the methods used to collect data? Yes 4. Is there congruity between the research methodology and the representation and analysis of data? Yes |
|  |  | (Continued) |
|  |  |  |
| Supplementary material 4 Continued | | |
| # | **Author, Tittle, Year of Publication** | **Critical appraisal ( Tool and its considerations)** |
|  |  | 1. Is there congruity between the research methodology and the interpretation of results? Yes 2. Is there a statement locating the researcher culturally or theoretically? Yes 3. Is the influence of the researcher on the research, and vice- versa, addressed? Not applicable 4. Are participants, and their voices, adequately represented? Yes 5. Is the research ethical according to current criteria or, for recent studies, and is there evidence of ethical approval by an appropriate body? Yes. (The document was not ethical approved by an appropriate body – Not applicable) 6. Do the conclusions drawn in the research report flow from the analysis, or interpretation, of the data? Yes   Overall appraisal: Include |
| 8 | Huong LTT, Hanh TTT, Toan LQ, Trang DTH, Quynh NT, Anh NQ, et al. Training need assessment for a master training program in Environmental Health program in Vietnam. AIMS Public Health. 2020;7(1):197-212. | Checklist for Analytical Cross-Sectional Study   1. Were the criteria for inclusion in the sample clearly defined? Not applicable (Inclusion criteria provided, exclusion not provided) 2. Were the study subjects and the setting described in detail? Yes. 3. Was the exposure measured in a valid and reliable way? Yes 4. Were objective, standard criteria used for measurement of the condition? Yes 5. Were confounding factors identified? Not applicable 6. Were strategies to deal with confounding factors stated? Not applicable 7. Were the outcomes measured in a valid and reliable way? Yes 8. Was appropriate statistical analysis used? Yes   Overall appraisal: Include |
| 9 | Purnell M. Findings from a training needs analysis survey to support health professionals across the research lifecycle. Health Info Libr J. 2020;37(2):118-27. | Checklist for Analytical Cross-Sectional Study   1. Were the criteria for inclusion in the sample clearly defined? Not applicable (authors selected six hospitals and 74 clinics) 2. Were the study subjects and the setting described in detail? Yes 3. Was the exposure measured in a valid and reliable way? Yes 4. Were objective, standard criteria used for measurement of the condition? Yes 5. Were confounding factors identified? Not applicable |
|  |  | (Continued) |
| Supplementary material 4 Continued | | |
| # | **Author, Tittle, Year of Publication** | **Critical appraisal ( Tool and its considerations)** |
|  |  | 1. Were strategies to deal with confounding factors stated? Not applicable 2. Were the outcomes measured in a valid and reliable way? Yes 3. Was appropriate statistical analysis used? Yes   Overall appraisal: Include |
| 10 | Bulawayo M, Silumbwe A, Munakampe MN, Mukumbuta N, Musabula J, Chewe M, et al. A needs assessment for postgraduate training in selected public health disciplines: evidence from health services organisations in Lusaka, Zambia. BMC Health Serv Res. 2020;20(1):1079. | Checklist for Analytical Cross-Sectional Study   1. Were the criteria for inclusion in the sample clearly defined? Not applicable 2. Were the study subjects and the setting described in detail? Yes 3. Was the exposure measured in a valid and reliable way? Yes 4. Were objective, standard criteria used for measurement of the condition? Yes 5. Were confounding factors identified? Not applicable 6. Were strategies to deal with confounding factors stated? Not applicable 7. Were the outcomes measured in a valid and reliable way? Yes 8. Was appropriate statistical analysis used? Yes   Overall appraisal: Include |
| 11 | Lee SA, Byth K, Gifford JA, Balasubramanian M, Fozzard CA, Skapetis T, et al. Assessment of Health Research Capacity in Western Sydney Local Health District (WSLHD): A Study on Medical, Nursing and Allied Health Professionals. J Multidiscip Healthc. 2020;13:153-63. | Checklist for Analytical Cross-Sectional Study   1. Were the criteria for inclusion in the sample clearly defined? Not applicable 2. Were the study subjects and the setting described in detail? yes 3. Was the exposure measured in a valid and reliable way? yes 4. Were objective, standard criteria used for measurement of the condition? yes 5. Were confounding factors identified? Not applicable 6. Were strategies to deal with confounding factors stated? Not applicable 7. Were the outcomes measured in a valid and reliable way? Yes 8. Was appropriate statistical analysis used? Yes   Overall appraisal: Include |
|  |  | (Continued) |
|  |  |  |
| Supplementary material 4 Continued | | |
| # | **Author, Tittle, Year of Publication** | **Critical appraisal ( Tool and its considerations)** |
|  | Workforce. J Public Health Manag Pract. 2019;25 Suppl 2, Public Health Workforce Interests and Needs Survey 2017:S16-S25. | 5. Were confounding factors identified? Not applicable  6 Were strategies to deal with confounding factors stated? Not applicable  7. Were the outcomes measured in a valid and reliable way? Yes  8. Was appropriate statistical analysis used? Yes  Overall appraisal: Include |
| 13 | O'Meara MG, Sobelson RK, Trigoso SM, Kramer RE, McNaughton C, Smartis RJ, et al. Ensuring a competent public health responder workforce: The CDC experience. J Emerg Manag. 2019;17(3):199-209. | Checklist for qualitative research   1. Is there congruity between the stated philosophical perspective and the research methodology? yes 2. Is there congruity between the research methodology and the research question or objectives? yes 3. Is there congruity between the research methodology and the methods used to collect data? yes 4. Is there congruity between the research methodology and the representation and analysis of data? yes 5. Is there congruity between the research methodology and the interpretation of results? yes 6. Is there a statement locating the researcher culturally or theoretically? yes 7. Is the influence of the researcher on the research, and vice- versa, addressed? Not applicable 8. Are participants, and their voices, adequately represented? Yes 9. Is the research ethical according to current criteria or, for recent studies, and is there evidence of ethical approval by an appropriate body? Not applicable 10. Do the conclusions drawn in the research report flow from the analysis, or interpretation, of the data? Yes.   Overall appraisal: Include |
| 14 | McKeever, J.Leider, J. P.Alford, A. A.Evans, D. Regional Training Needs Assessment: A First Look at High-Priority Training Needs Across the United States by Region. J Public Health Manag Pract. 2019;25 Suppl 2, | Checklist for Analytical Cross-Sectional Study   1. Were the criteria for inclusion in the sample clearly defined? Not applicable (Inclusion criteria provided, exclusion not provided) 2. Were the study subjects and the setting described in detail? Yes. 3. Was the exposure measured in a valid and reliable way? Yes 4. Were objective, standard criteria used for measurement of the condition? Yes |
|  |  | (Continued) |
|  |  |  |
| Supplementary material 4 Continued | | |
| # | **Author, Tittle, Year of Publication** | **Critical appraisal ( Tool and its considerations)** |
|  | Public Health Workforce Interests and Needs Survey 2017: S166-S176 | 1. Were confounding factors identified? Not applicable 2. Were strategies to deal with confounding factors stated? Not applicable 3. Were the outcomes measured in a valid and reliable way? Yes 4. Was appropriate statistical analysis used? Yes   Overall appraisal: Include |
| 15 | Bogaert K, Castrucci BC, Gould E, Rider N, Whang C, Corcoran E. Top Training Needs of the Governmental Public Health Workforce. J Public Health Manag Pract. 2019;25 Suppl 2, Public Health Workforce Interests and Needs Survey 2017:S134-S44. | Checklist for Analytical Cross-Sectional Study   1. Were the criteria for inclusion in the sample clearly defined? Not applicable 2. Were the study subjects and the setting described in detail? Yes. 3. Was the exposure measured in a valid and reliable way? Yes 4. Were objective, standard criteria used for measurement of the condition? Yes 5. Were confounding factors identified? Not applicable 6. Were strategies to deal with confounding factors stated? Not applicable 7. Were the outcomes measured in a valid and reliable way? Yes 8. Was appropriate statistical analysis used? Yes   Overall appraisal: Include |
| 16 | Yeager, V. A.Wisniewski, J. M. Chapple-McGruder, T.Castrucci, B. C.Gould, E. Public Health Workforce Self-Identified Training Needs by Jurisdiction and Job Type. J Public Health Manag Pract.2019; 25 (2):181-190 | Checklist for Analytical Cross-Sectional Study   1. Were the criteria for inclusion in the sample clearly defined? Not applicable 2. Were the study subjects and the setting described in detail? Yes. 3. Was the exposure measured in a valid and reliable way? Yes 4. Were objective, standard criteria used for measurement of the condition? Yes 5. Were confounding factors identified? Not applicable 6. Were strategies to deal with confounding factors stated? Not applicable 7. Were the outcomes measured in a valid and reliable way? Yes 8. Was appropriate statistical analysis used? Yes   Overall appraisal: Include |
| 17 | Arogundade, L. Akinwumi, T. Molemodile, S. Nwaononiwu, E. Ezika, J. Yau, I , et al. | Mixed Methods Appraisal Tool (MMAT), version 2018  S1. Are there clear research questions? Yes  S2. Do the collected data allow to address the research questions? Yes |
|  |  | (Continued) |
| Supplementary material 4 Continued | | |
| # | **Author, Tittle, Year of Publication** | **Critical appraisal ( Tool and its considerations)** |
|  | Lessons from a training needs assessment to strengthen the capacity of routine immunization service providers in Nigeria. BMC Health Serv Res.2019;19 (1):664. | 1-4 Quantitative randomized controlled trials, Quantitative randomized controlled trials and Quantitative randomized controlled trials: Comments Not applicable, as authors used a mix-methods approach.  5. Mixed methods  5.1. Is there an adequate rationale for using a mixed methods design to address the research question? Yes  5.2. Are the different components of the study effectively integrated to answer the research question? Yes  5.3. Are the outputs of the integration of qualitative and quantitative components adequately interpreted? Yes  5.4. Are divergences and inconsistencies between quantitative and qualitative results adequately addressed? Yes  5.5. Do the different components of the study adhere to the quality criteria of each tradition of the methods involved? Yes  Overall appraisal: Include |
| 18 | Joly, B. M.Coronado, F.Bickford, B. C.Leider, J. P.Alford, A.McKeever, J. A Review of Public Health Training Needs Assessment Approaches: Opportunities to Move Forward. J Public Health Manag Pract. 2018; 24 (6): 571-577 | No robust methods… We should eliminate it…. The methods did not allow to determine the process to obtain the 24 surveys for TNA.  The tittle “A Review of Public Health Training Needs Assessment Approaches:”. So it is supposed that the article is a review.   1. Is the review question clearly and explicitly stated? Yes 2. Were the inclusion criteria appropriate for the review question? Unclear ( authors provided inclusion criteria, but not exclusion criteria) 3. Was the search strategy appropriate? Unclear ( authors mentioned that a review was done about this topic, but they did not stated the search strategy) 4. Were the sources and resources used to search for studies adequate? Yes 5. Were the criteria for appraising studies appropriate? Unclear. 6. Was critical appraisal conducted by two or more reviewers independently? Unclear. 7. Were there methods to minimize errors in data extraction? Unclear 8. Were the methods used to combine studies appropriate? Unclear 9. Was the likelihood of publication bias assessed? Unclear |
|  |  | (Continued) |
|  |  |  |
| Supplementary material 4 Continued | | |
| # | **Author, Tittle, Year of Publication** | **Critical appraisal ( Tool and its considerations)** |
|  |  | 10. Were recommendations for policy and/or practice supported by the reported data? Yes  11. Were the specific directives for new research appropriate? Yes  Overall appraisal: Include |
| 19 | Mid-Atlantic Regional Public Health Training Center (MAR-PHTC). Public Health Training Needs Assessment: Report on Maryland Department of Health and Mental Hygiene.2017. Available from Training Needs Assessment Report - Maryland DHMH.pdf Accessed on 24^th^ March 2022. | Toolkit: Qualitative research   1. Is there congruity between the stated philosophical perspective and the research methodology? Yes 2. Is there congruity between the research methodology and the research question or objectives? Yes 3. Is there congruity between the research methodology and the methods used to collect data? Yes 4. Is there congruity between the research methodology and the representation and analysis of data? Yes 5. Is there congruity between the research methodology and the interpretation of results? Yes 6. Is there a statement locating the researcher culturally or theoretically? Yes 7. Is the influence of the researcher on the research, and vice- versa, addressed? Not applicable 8. Are participants, and their voices, adequately represented? Yes 9. Is the research ethical according to current criteria or, for recent studies, and is there evidence of ethical approval by an appropriate body? Yes. (The document was not ethical approved by an appropriate body – Not applicable) 10. Do the conclusions drawn in the research report flow from the analysis, or interpretation, of the data? Yes   Overall appraisal: Include |
| 20 | Chapple-McGruder, T.Leider, J. P. Beck, A. J.Castrucci, B. C.Harper, E. Sellers, K. Examining state health agency epidemiologists and their training needs. Ann Epidemiol. 2017;27(2): 83-88 | Checklist for Analytical Cross-Sectional Study   1. Were the criteria for inclusion in the sample clearly defined? Yes 2. Were the study subjects and the setting described in detail? Yes. 3. Was the exposure measured in a valid and reliable way? Yes 4. Were objective, standard criteria used for measurement of the condition? Yes 5. Were confounding factors identified? Yes |
|  |  | (Continued) |
| Supplementary material 4 Continued | | |
| # | **Author, Tittle, Year of Publication** | **Critical appraisal ( Tool and its considerations)** |
|  |  | 6. Were strategies to deal with confounding factors stated? Yes  7. Were the outcomes measured in a valid and reliable way? Yes  8. Was appropriate statistical analysis used? Yes  Overall appraisal: Include |
| 21 | Kornfeld, J.Sznol, J.Lee, D. Characterizing the Business Skills of the Public Health Workforce: Practical Implications From the Public Health Workforce Interests and Needs Survey (PH WINS). J Public Health Manag Pract. 2015; 21 Suppl 6: S159-67 | Checklist for Analytical Cross-Sectional Study   1. Were the criteria for inclusion in the sample clearly defined? Not applicable 2. Were the study subjects and the setting described in detail? Yes 3. Was the exposure measured in a valid and reliable way? Yes 4. Were objective, standard criteria used for measurement of the condition? Yes 5. Were confounding factors identified? Not applicable 6. Were strategies to deal with confounding factors stated? Not applicable 7. Were the outcomes measured in a valid and reliable way? Yes 8. Was appropriate statistical analysis used? Yes   Overall appraisal: Include |
| 22 | Ye, J. Leep, C.Robin, N.Newman, S. Perception of Workforce Skills Needed Among Public Health Professionals in Local Health Departments: Staff Versus Top Executives. J Public Health Manag Pract.2015; 21 Suppl 6:S151-8 | Checklist for Analytical Cross-Sectional Study   1. Were the criteria for inclusion in the sample clearly defined? Not applicable 2. Were the study subjects and the setting described in detail? Yes 3. Was the exposure measured in a valid and reliable way? Yes 4. Were objective, standard criteria used for measurement of the condition? Yes 5. Were confounding factors identified? Not applicable 6. Were strategies to deal with confounding factors stated? Not applicable 7. Were the outcomes measured in a valid and reliable way? Yes 8. Was appropriate statistical analysis used? Yes   Overall appraisal: Include |
| 23 | Dixon, B. E.McFarlane, T. D.Dearth, S. Grannis, S. J.Gibson, P. J. Characterizing Informatics Roles and Needs of Public Health Workers: Results From the Public Health Workforce Interests and Needs Survey. J Public Health Manag Pract.2015; 21 Suppl 6: S130-40 | Checklist for Analytical Cross-Sectional Study   1. Were the criteria for inclusion in the sample clearly defined? Not applicable 2. Were the study subjects and the setting described in detail? Yes 3. Was the exposure measured in a valid and reliable way? Yes 4. Were objective, standard criteria used for measurement of the condition? Yes 5. Were confounding factors identified? Not applicable 6. Were strategies to deal with confounding factors stated? Not applicable |
|  |  | (Continued) |
|  |  |  |
|  |  |  |
| Supplementary material 4 Continued | | |
| # | **Author, Tittle, Year of Publication** | **Critical appraisal ( Tool and its considerations)** |
|  |  | 7. Were the outcomes measured in a valid and reliable way? Yes  8. Was appropriate statistical analysis used? Yes  Overall appraisal: Include |
| 24 | Sellers, K.Leider, J. P.Harper, E. Castrucci, B. C.Bharthapudi, K. Liss-Levinson, R, et al. The Public Health Workforce Interests and Needs Survey: The First National Survey of State Health Agency Employees. J Public Health Manag Pract. 2015; 21 Suppl 6: S13-27. | Checklist for Analytical Cross-Sectional Study   1. Were the criteria for inclusion in the sample clearly defined? Not applicable 2. Were the study subjects and the setting described in detail? Yes 3. Was the exposure measured in a valid and reliable way? Yes 4. Were objective, standard criteria used for measurement of the condition? Yes 5. Were confounding factors identified? Not applicable 6. Were strategies to deal with confounding factors stated? Not applicable 7. Were the outcomes measured in a valid and reliable way? Yes 8. Was appropriate statistical analysis used? Yes   Overall appraisal: Include |
| 25 | Bloomington Public Health .Staff Training Needs Assessment Bloomington Public Health. 2014.Available from: BPH Training Needs Assessment Report (naccho.org) Accessed on Accessed on 24^th^ March 2022 | Checklist for Analytical Cross-Sectional Study   1. Were the criteria for inclusion in the sample clearly defined? Not applicable (each worker was included) 2. Were the study subjects and the setting described in detail? Yes 3. Was the exposure measured in a valid and reliable way? Yes 4. Were objective, standard criteria used for measurement of the condition? Yes 5. Were confounding factors identified? Not applicable 6. Were strategies to deal with confounding factors stated? Not applicable 7. Were the outcomes measured in a valid and reliable way? Yes 8. Was appropriate statistical analysis used? Yes   Overall appraisal: Include |
| 26 | Adkoli, B. V.Al-Umran, K. U. Al-Sheikh, M. H.Deepak, K. K. Innovative method of needs assessment for faculty development programs in a Gulf medical | Checklist for Analytical Cross-Sectional Study   1. Were the criteria for inclusion in the sample clearly defined? Not applicable (each faculty member was included) 2. Were the study subjects and the setting described in detail? Yes 3. Was the exposure measured in a valid and reliable way? Yes |
|  |  | (Continued) |
| Supplementary material 4 Continued | | |
| # | **Author, Tittle, Year of Publication** | **Critical appraisal ( Tool and its considerations)** |
|  | school. Educ Health (Abingdon).2010;23(3): 389. | 4. Was the exposure measured in a valid and reliable way? Yes  5. Were confounding factors identified? Not applicable  6. Were strategies to deal with confounding factors stated? Not applicable  7. Were the outcomes measured in a valid and reliable way? Yes  8. Was appropriate statistical analysis used? Yes  Overall appraisal: Include |
| 27 | Hites, L. S. Lafreniere, A. V. Wingate, M. S. Anderson, A. C. Ginter, P. M.Santacaterina, L, et al. Expanding the public health emergency preparedness competency set to meet specialized local and evolving national needs: a needs assessment and training approach. J Public Health Manag Pract.2007; 13(5): 497-505 | Checklist for text and opinions   1. Is the source of the opinion clearly identified? Yes 2. Does the source of opinion have standing in the field of expertise? Yes 3. Are the interests of the relevant population the central focus of the opinion? Yes 4. Is the stated position the result of an analytical process, and is there logic in the opinion expressed? Yes 5. Is there reference to the extant literature? Yes 6. Is any incongruence with the literature/sources logically defended? Yes   Overall appraisal: Include |
| 28 | Dato, V. M. Potter, M. A. Fertman, C. I. Training readiness of public health agencies: a framework for assessment. J Public Health Manag Pract.2001; 7(4): 91-5. | Checklist for qualitative research   1. Is there congruity between the stated philosophical perspective and the research methodology? Yes 2. Is there congruity between the research methodology and the research question or objectives? Yes 3. Is there congruity between the research methodology and the methods used to collect data? Yes 4. Is there congruity between the research methodology and the representation and analysis of data? Yes 5. Is there congruity between the research methodology and the interpretation of results? Yes 6. Is there a statement locating the researcher culturally or theoretically? Yes 7. Is the influence of the researcher on the research, and vice- versa, addressed? Not applicable 8. Are participants, and their voices, adequately represented? Yes |
|  |  | (Continued) |
|  |  |  |
|  |  |  |
|  |  |  |
| Supplementary material 4 Continued | | |
| # | **Author, Tittle, Year of Publication** | **Critical appraisal ( Tool and its considerations)** |
|  |  | 9. Is the research ethical according to current criteria or, for recent studies, and is there evidence of ethical approval by an appropriate body? Yes  10. Do the conclusions drawn in the research report flow from the analysis, or interpretation, of the data? Yes  Overall appraisal: Include |
| 29 | Ruck, N. F.Hafeez, M. Manzur, S. Nasir, F. Rivers, K. Prozesky, D. Assessing management training needs: a study in the Punjab health services, Pakistan. J Health Popul Dev Ctries.1999;2 (1): 78-87 | Checklist for qualitative research   1. Is there congruity between the stated philosophical perspective and the research methodology? Yes 2. Is there congruity between the research methodology and the research question or objectives? Yes 3. Is there congruity between the research methodology and the methods used to collect data? Yes 4. Is there congruity between the research methodology and the representation and analysis of data? Yes 5. Is there congruity between the research methodology and the interpretation of results? Yes 6. Is there a statement locating the researcher culturally or theoretically? Yes 7. Is the influence of the researcher on the research, and vice- versa, addressed? Not applicable 8. Are participants, and their voices, adequately represented? Yes 9. Is the research ethical according to current criteria or, for recent studies, and is there evidence of ethical approval by an appropriate body? Yes 10. Do the conclusions drawn in the research report flow from the analysis, or interpretation, of the data? Yes   Overall appraisal: Include |
| 30 | **Western Region** Public Health Training Center. Needs Assessment. Available from: Needs Assessment \| Western Region Public | Checklist for text and opinions   1. Is the source of the opinion clearly identified? Yes 2. Does the source of opinion have standing in the field of expertise? Yes |
|  |  | (Continued) |
|  |  |  |
|  |  |  |
|  |  |  |
| Supplementary material 4 Continued | | |
| # | **Author, Tittle, Year of Publication** | **Critical appraisal ( Tool and its considerations)** |
|  | Health Training Center (arizona.edu) Accessed on 29^th^ April 2022 | 3. Are the interests of the relevant population the central focus of the opinion? Yes  4. Is the stated position the result of an analytical process, and is there logic in the opinion expressed? Not applicable  5. Is there reference to the extant literature? Not applicable  6. Is any incongruence with the literature/sources logically defended? Not applicable  Overall appraisal: Include |
| 31 | Academy for Educational Development. Support for Croatia’s non-governmental Sector CroNGO: Organizational Capacity Self-Assessment Tool Guidelines for Developing an Organizational Training Plan. Available from: Organizational Capacity Self-Assessment Tool.pdf (ngoconnect.net) Accessed on 29^th^ April 2022 | Checklist for text and opinions   1. Is the source of the opinion clearly identified? Yes 2. Does the source of opinion have standing in the field of expertise? Yes 3. Are the interests of the relevant population the central focus of the opinion? Yes 4. Is the stated position the result of an analytical process, and is there logic in the opinion expressed? Not applicable 5. Is there reference to the extant literature? Not applicable 6. Is any incongruence with the literature/sources logically defended? Not applicable   Overall appraisal: Include |
| 32 | First Nations Health Managers Association. The First Nations Health Manager Competency Framework Self-Assessment Tool | Checklist for text and opinions   1. Is the source of the opinion clearly identified? Yes 2. Does the source of opinion have standing in the field of expertise? Yes 3. Are the interests of the relevant population the central focus of the opinion? Yes 4. Is the stated position the result of an analytical process, and is there logic in the opinion expressed? Not applicable 5. Is there reference to the extant literature? Not applicable 6. Is any incongruence with the literature/sources logically defended? Not applicable   Overall appraisal: Include |
| 33 | National Association of County and City Health Officials. 2018 Forces of Change. | Checklist for text and opinions   1. Is the source of the opinion clearly identified? Yes |
|  |  | (Continued) |
|  |  |  |
|  |  |  |
|  |  |  |
| Supplementary material 4 Continued | | |
| # | **Author, Tittle, Year of Publication** | **Critical appraisal ( Tool and its considerations)** |
|  |  | 2. Does the source of opinion have standing in the field of expertise? Yes  3. Are the interests of the relevant population the central focus of the opinion? Yes  4. Is the stated position the result of an analytical process, and is there logic in the opinion expressed? Not applicable  5. Is there reference to the extant literature? Not applicable  6. Is any incongruence with the literature/sources logically defended? Not applicable  Overall appraisal: Include |
| 34 | GOV.UK. Public health skills and knowledge framework: tools and guidance. | Checklist for text and opinions   1. Is the source of the opinion clearly identified? Yes 2. Does the source of opinion have standing in the field of expertise? Yes 3. Are the interests of the relevant population the central focus of the opinion? Yes 4. Is the stated position the result of an analytical process, and is there logic in the opinion expressed? Not applicable 5. Is there reference to the extant literature? Not applicable 6. Is any incongruence with the literature/sources logically defended? Not applicable   Overall appraisal: Include |
| 35 | NHS Leadership Academic. Clinical Leadership Competency Framework: Self-assessment tool. 2012. | Checklist for text and opinions   1. Is the source of the opinion clearly identified? Yes 2. Does the source of opinion have standing in the field of expertise? Yes 3. Are the interests of the relevant population the central focus of the opinion? Yes 4. Is the stated position the result of an analytical process, and is there logic in the opinion expressed? Not applicable 5. Is there reference to the extant literature? Not applicable 6. Is any incongruence with the literature/sources logically defended? Not applicable   Overall appraisal: Include |

**Supplementary material 5: Literature that has been included in this review.**

1. Kulik PKG, Leider JP, Beck AJ. Leadership Perspectives on Local Health Department Workforce Development: A Regional Training Needs Assessment. J Public Health Manag Pract. 2022;28(2):E619-E23.
2. Daly ER, Gendelman M, Arrazola J, Sellers K. Training Needs and Awareness of Emerging Areas of Public Health Practice Among Epidemiologists Working in State Health Agency Central Offices in the United States, 2017. J Public Health Manag Pract. 2022;28(1):E273-E82.
3. Sainkhuu S, Cunha-Cruz J, Rogers M, Knerr S, Bekemeier B. Evaluation of Training Gaps Among Public Health Practitioners in Washington State. J Public Health Manag Pract. 2021;27(5):473-83.
4. Taylor HL, Yeager VA. Core Competency Gaps Among Governmental Public Health Employees With and Without a Formal Public Health Degree. J Public Health Manag Pract. 2021;27(1):20-9.
5. Kalbarczyk A, Rao A, Alonge O. A mixed methods study to develop a tool to assess institutional readiness to conduct knowledge translation activities in low-income and middle-income countries. BMJ Open. 2021;11(10):e050049.
6. Markaki A, Malhotra S, Billings R, Theus L. Training needs assessment: tool utilization and global impact. BMC Med Educ. 2021;21(1):310.
7. Region V Public Health Training Center. 2020 Training Needs Assessment Survey.2020. Available from Training Needs Assessment_2020_RVPHTC.pdf - Google Drive Accessed on 9^th^ July 2023
8. Huong LTT, Hanh TTT, Toan LQ, Trang DTH, Quynh NT, Anh NQ, et al. Training need assessment for a master training program in Environmental Health program in Vietnam. AIMS Public Health. 2020;7(1):197-212.
9. Purnell M. Findings from a training needs analysis survey to support health professionals across the research lifecycle. Health Info Libr J. 2020;37(2):118-27.
10. Bulawayo M, Silumbwe A, Munakampe MN, Mukumbuta N, Musabula J, Chewe M, et al. A needs assessment for postgraduate training in selected public health disciplines: evidence from health services organisations in Lusaka, Zambia. BMC Health Serv Res. 2020;20(1):1079.
11. Lee SA, Byth K, Gifford JA, Balasubramanian M, Fozzard CA, Skapetis T, et al. Assessment of Health Research Capacity in Western Sydney Local Health District (WSLHD): A Study on Medical, Nursing and Allied Health Professionals. J Multidiscip Healthc. 2020;13:153-63.
12. Bogaert K, Castrucci BC, Gould E, Sellers K, Leider JP, Whang C, et al. The Public Health Workforce Interests and Needs Survey (PH WINS 2017): An Expanded Perspective on the State Health Agency Workforce. J Public Health Manag Pract. 2019;25 Suppl 2, Public Health Workforce Interests and Needs Survey 2017:S16-S25.
13. O'Meara MG, Sobelson RK, Trigoso SM, Kramer RE, McNaughton C, Smartis RJ, et al. Ensuring a competent public health responder workforce: The CDC experience. J Emerg Manag. 2019;17(3):199-209.
14. McKeever, J.Leider, J. P.Alford, A. A.Evans, D. Regional Training Needs Assessment: A First Look at High-Priority Training Needs Across the United States by Region. J Public Health Manag Pract. 2019;25 Suppl 2, Public Health Workforce Interests and Needs Survey 2017: S166-S176
15. Bogaert K, Castrucci BC, Gould E, Rider N, Whang C, Corcoran E. Top Training Needs of the Governmental Public Health Workforce. J Public Health Manag Pract. 2019;25 Suppl 2, Public Health Workforce Interests and Needs Survey 2017:S134-S44.
16. Yeager, V. A.Wisniewski, J. M. Chapple-McGruder, T.Castrucci, B. C.Gould, E. Public Health Workforce Self-Identified Training Needs by Jurisdiction and Job Type. J Public Health Manag Pract.2019; 25 (2):181-190
17. Arogundade, L. Akinwumi, T. Molemodile, S. Nwaononiwu, E.
    Ezika, J. Yau, I , et al. Lessons from a training needs assessment to strengthen the capacity of routine immunization service providers in Nigeria. BMC Health Serv Res.2019;19 (1):664.
18. Joly, B. M.Coronado, F.Bickford, B. C.Leider, J. P.Alford, A.McKeever, J. A Review of Public Health Training Needs Assessment Approaches: Opportunities to Move Forward. J Public Health Manag Pract. 2018; 24 (6): 571-577
19. Mid-Atlantic Regional Public Health Training Center (MAR-PHTC). Public Health Training Needs Assessment: Report on Maryland Department of Health and Mental Hygiene.2017. Available from Training Needs Assessment Report - Maryland DHMH.pdf Accessed on 9^th^ July 2023
20. Chapple-McGruder, T.Leider, J. P.Beck, A. J.Castrucci, B. C.Harper, E. Sellers, K. Examining state health agency epidemiologists and their training needs. Ann Epidemiol. 2017;27(2): 83-88
21. Kornfeld, J.Sznol, J.Lee, D. Characterizing the Business Skills of the Public Health Workforce: Practical Implications From the Public Health Workforce Interests and Needs Survey (PH WINS). J Public Health Manag Pract. 2015; 21 Suppl 6: S159-67
22. Ye, J. Leep, C.Robin, N.Newman, S. Perception of Workforce Skills Needed Among Public Health Professionals in Local Health Departments: Staff Versus Top Executives. J Public Health Manag Pract.2015; 21 Suppl 6:S151-8
23. Dixon, B. E.McFarlane, T. D.Dearth, S. Grannis, S. J.Gibson, P. J. Characterizing Informatics Roles and Needs of Public Health Workers: Results From the Public Health Workforce Interests and Needs Survey. J Public Health Manag Pract.2015; 21 Suppl 6: S130-40
24. Sellers, K.Leider, J. P.Harper, E. Castrucci, B. C.Bharthapudi, K.
    Liss-Levinson, R, et al. The Public Health Workforce Interests and Needs Survey: The First National Survey of State Health Agency Employees. J Public Health Manag Pract. 2015; 21 Suppl 6: S13-27.
25. Bloomington Public Health .Staff Training Needs Assessment Bloomington Public Health. 2014.Available from: BPH Training Needs Assessment Report (naccho.org) Accessed on 9^th^ July 2023
26. Adkoli, B. V.Al-Umran, K. U.Al-Sheikh, M. H.Deepak, K. K. Innovative method of needs assessment for faculty development programs in a Gulf medical school. Educ Health (Abingdon).2010;23(3): 389.
27. Hites, L. S. Lafreniere, A. V. Wingate, M. S. Anderson, A. C. Ginter, P. M.Santacaterina, L, et al. Expanding the public health emergency preparedness competency set to meet specialized local and evolving national needs: a needs assessment and training approach. J Public Health Manag Pract.2007; 13(5): 497-505
28. Dato, V. M. Potter, M. A. Fertman, C. I. Training readiness of public health agencies: a framework for assessment. J Public Health Manag Pract.2001; 7(4): 91-5.
29. Ruck, N. F.Hafeez, M. Manzur, S. Nasir, F. Rivers, K. Prozesky, D. Assessing management training needs: a study in the Punjab health services, Pakistan. J Health Popul Dev Ctries.1999;2 (1): 78-87
30. **Western Region.** Public Health Training Center. Needs Assessment. Available from: Needs Assessment | Western Region Public Health Training Center (arizona.edu) Accessed on 9^th^ July 2023
31. Academy for Educational Development. Support for Croatia’s non-governmental Sector CroNGO: Organizational Capacity SelfAssessment Tool Guidelines for Developing an Organizational Training Plan. Available from: Organizational Capacity Self-Assessment Tool.pdf (ngoconnect.net) Accessed on 9^th^ July 2023
32. First Nations Health Managers Association. The First Nations Health Manager Competency Framework Self-Assessment Tool. 2019. Available from: PowerPoint Presentation (fnhmaconference.ca) Accessed on 9^th^ July 2023
33. National Association of County and City Health Officials. 2018 Forces of Change. 2018. Available from: https://www.naccho.org/uploads/downloadable-resources/2018-Forces-of-Change_March-2018f.pdf Accessed on 9^th^ July 2023
34. GOV.UK. Public health skills and knowledge framework: tools and guidance. 2018.Available from: PHSKF: self-assessment tool. Accessed on 9^th^ July 2023
35. NHS Leadership Academic. Clinical Leadership Competency Framework: Self assessment tool. 2012. Available from: CLCF Self Assessment_Layout 1 (leadershipacademy.nhs.uk) Accessed on 9^th^ July 2023
36. American Nurses Association. ANA LEADERSHIP COMPETENCY MODEL. 2018. Available from: 177626 ANA Leadership Booklet 01 (nursingworld.org) Accessed on 9^th^ July 2023
37. American Organization of Nurse Executives. AONE Nurse Executive Competencies. 2015. Available from: https://www.aonl.org/sites/default/files/aone/nec.pdf Accessed on 9^th^ July 2023
38. American Organization of Nurse Executives. AONE Nurse Executive Competencies. 2005. Available from: https://www.nurseleader.com/action/showPdf?pii=S1541-4612%2805%2900007-8 Accessed on 9^th^ July 2023

**Supplementary material 6** PRISMA 2020 Flow Diagram: Training needs assessment tools for public health workforce at institutional and individual level - a review

**Identification of studies via other methods**

**Identification of studies via databases and registers**

Records identified from:

Google: handsearching (n=100)

Consultation (n =13)

Records identified from:

PubMed (n = 11 425)

Google scholar (n = 569)

**Identification**

Records excluded

(n = 11 917)

Records screened by tittle

(n = 11 994)

Reports excluded:

Duplicated (n = 5)

Link not working (n =2)

Eligibility criteria (n = 93)

Reports screened for eligibility

(n = 113)

**Screening**

Reports screened by abstract and full text

(n = 77)

Reports excluded:

Full text (n = 3)

Eligibility criteria (n=49)

Studies included in review

(n = 38)

**Included**

| Supplementary material 7 Extraction information: Training needs assessment tools for public health workforce at institutional and individual level - a review | | | | | | | |
| --- | --- | --- | --- | --- | --- | --- | --- |
| # | **Vancouver citation** | **Country/location**  **and Organisation** | **Workforce studied.** | **Objective of assessment**  **(Organisational, individual)** | **Assessment Tool’s name and methods of development** | **Methods used for the assessment** | **Tool: Availability** |
| 1 | Kulik PKG, Leider JP, Beck AJ. Leadership Perspectives on Local Health Department Workforce Development: A Regional Training Needs Assessment. J Public Health Manag Pract. 2022;28(2):E619-E23. | USA  Illinois,  Indiana,  Michigan, Minnesota,  Ohio, and Wisconsin.  Small Local Health Departments ( LHDs) | Regional local governmental public health workforce | Workforce development needs of public health professionals  Organisational | -Region V Public Health Training Center Training Needs Assessment.  -The instrument was developed using previous work from Public Health Workforce Interests and Needs Survey( PH WINS) and Directorʼs Assessment of Workforce Needs Survey (DAWNS). | Survey | jphmp_2021_05_26_kulik_2100143_sdc1.pdf (lww.com) |
| 2 | Daly ER, Gendelman M, Arrazola J, Sellers K. Training Needs and Awareness of Emerging Areas of Public Health Practice Among Epidemiologists Working in State Health Agency Central Offices in the United States, 2017. J Public Health Manag Pract. 2022;28(1):E273-E82. | USA  State Health Agency Central Offices (SHA-CO) | Epidemiologist | Training needs self-assessment | Authors selected 2017 PH WINS.  PH WINS ‘Methods: Leider JP, Bharthapudi K, Pineau V, Liu L, Harper E. The Methods Behind PH WINS. J Public Health Manag Pract. 2015;21 Suppl 6:S28-35. | Survey | Tool: Public Health Workforce Interests and Needs Survey - State (debeaumont.org)  Codebook  PH-WINS-2017-codebook_annotated_externalresearchers.pdf (debeaumont.org) |
|  |  |  |  |  |  |  | (Continued) |
| Supplementary material 7 Continued | | | | | | | |
| # | **Vancouver citation** | **Country/location**  **and Organisation** | **Workforce studied.** | **Objective of assessment**  **(Organisational, individual)** | **Assessment Tool’s name and methods of development** | **Methods used for the assessment** | **Tool: Availability** |
|  |  |  |  |  | The Methods Behind PH WINS - PubMed (nih.gov) |  |  |
| 3 | Sainkhuu S, Cunha-Cruz J, Rogers M, Knerr S, Bekemeier B. Evaluation of Training Gaps Among Public Health Practitioners in Washington State. J Public Health Manag Pract. 2021;27(5):473-83. | USA  Washington State  Northwest Center for Public Health Practice (NWCPHP) | Public Health Practitioners, local health departments | Training needs self-assessment | no name.  Based on prior NWCPHP training needs surveys used in 2010 and 2013.  2 domains were renamed to simplify language and provide clarity. | Survey | Tool not available. |
| 4 | Taylor HL, Yeager VA. Core Competency Gaps Among Governmental Public Health Employees With and Without a Formal Public Health Degree. J Public Health Manag Pract. 2021;27(1):20-9. | USA  Governmental public health | U.S. state and local governmental public health workers. | Training needs self-assessment | Authors selected 2017 PH WINS.  PH WINS ‘Methods: Leider JP, Bharthapudi K, Pineau V, Liu L, Harper E. The Methods Behind PH WINS. J Public Health Manag Pract. 2015;21 Suppl 6:S28-35.  The Methods Behind PH WINS - PubMed (nih.gov) | Survey | Tool: Public Health Workforce Interests and Needs Survey - State (debeaumont.org)  Codebook  PH-WINS-2017-codebook_annotated_externalresearchers.pdf (debeaumont.org) |
|  |  |  |  |  |  |  | (Continued) |
| Supplementary material 7 Continued | | | | | | | |
| # | **Vancouver citation** | **Country/location**  **and Organisation** | **Workforce studied.** | **Objective of assessment**  **(Organisational, individual)** | **Assessment Tool’s name and methods of development** | **Methods used for the assessment** | **Tool: Availability** |
| 5 | Kalbarczyk A, Rao A, Alonge O. A mixed methods study to develop a tool to assess institutional readiness to conduct knowledge translation activities in low-income and middle-income countries. BMJ Open. 2021;11(10):e050049. | Bangladesh, Democratic Republic of the Congo, Ethiopia, India,  Indonesia and Nigeria  six academic STRIPE institutions (James P Grant School of Public Health, BRAC University, School of Public Health, University of Kinshasa, College of Health Sciences, Addis Ababa University, Indian Institute of Health Management Research University Faculty of Medicine, Public Health, and Nursing, Gadjah Mada University, College of Medicine, University of Ibadan | Academic institutions’ staff.  Synthesis and  Translation of Research and Innovations from Polio Eradication’ (STRIPE) | Training needs self-assessment  and organisational | -Assessing and Enhancing the Readiness of Academic Institutions in Low- income and middle-income countries(LMICs) to Conduct Knowledge Translation Activities  -Step 1:A literature review.  -Step 2: stakeholder interviews  -Step 3: quantitative survey | Survey | Knowledge Translation Survey - Wider Community (bmj.com) |
|  |  |  |  |  |  |  | (Continued) |
| Supplementary material 7 Continued | | | | | | | |
| # | **Vancouver citation** | **Country/location**  **and Organisation** | **Workforce studied.** | **Objective of assessment**  **(Organisational, individual)** | **Assessment Tool’s name and methods of development** | **Methods used for the assessment** | **Tool: Availability** |
| 6 | Markaki A, Malhotra S, Billings R, Theus L. Training needs assessment: tool utilization and global impact. BMC Med Educ. 2021;21(1):310. | Australia  Greece  Ireland  New Zealand  Poland  Singapore  UK  USA  Bulgaria  India  Indonesia  Nigeria  Saint Lucia  South Africa  Tanzania  Turkey  Sudan | Nurses  Physicians  Other healthcare professionals  Health insurance employees | Training needs self-assessment  and organisational | The Hennessy-Hicks Training Needs Analysis questionnaire  Methods: literature, focus groups and semi-structured interviews.  (3) HENNESSY-HICKS TRAINING NEEDS ANALYSIS QUESTIONNAIRE AND MANUAL For use at a local level to identify training and development needs \| veenu arora - Academia.edu | Questionnaire |  |
| 7 | Region V Public Health Training Center. 2020 Training Needs Assessment Survey.2020. Available from Training Needs Assessment_2020_RVPHTC.pdf - Google Drive Accessed on 9^th^ July 2023 | USA  Illinois,  Indiana,  Michigan,  Minnesota,  Ohio, and  Wisconsin. | LHD staff in our region | Training needs self-assessment | - “Survey Tool Training Needs Assessment: The Region V Public Health Training Center (RVPHTC)”  -Survey items adapted elements of several existing sources: the Directors Assessment of | Survey | Training Needs Assessment_2020_RVPHTC.pdf - Google Drive (Appendix) |
|  |  |  |  |  |  |  | (Continued) |
| Supplementary material 7 Continued | | | | | | | |
| # | **Vancouver citation** | **Country/location**  **and Organisation** | **Workforce studied.** | **Objective of assessment**  **(Organisational, individual)** | **Assessment Tool’s name and methods of development** | **Methods used for the assessment** | **Tool: Availability** |
|  |  | Region V Public Health Training Center |  |  | Workforce Needs Survey (DAWNS), the Public Health Workforce Interests and Needs Survey (PH WINS), and other assessments previously implemented in the region |  |  |
| 8 | Huong LTT, Hanh TTT, Toan LQ, Trang DTH, Quynh NT, Anh NO, et al. Training need assessment for a master training program in Environmental Health program in Vietnam . AIMS Public Health. 2020;7(1):197-212. | Vietnam  Dien Bien,  Hanoi,  Ha Tinh,  Binh Dinh,  Dak Lak, Hochiminh city and Can Tho.  Hanoi University of Public Health | experts from universities, managers and  staff national level experts from health, environment, agriculture and rural development sectors at provincial level | Training needs self-assessment  and organisational assessment | - No name  - This cross-sectional study used a mixed-methods approach with qualitative and quantitative components. The authors did not mention how these methods were created. | A qualitative survey.  - Self-administered questionnaires. | Tool not available  in-depth interviews  -Focus group.  -Delphi techniques. |
| 9 | Purnell M. Findings from a training needs analysis survey to support health professionals across the | Australia | Administration, Allied Health, Doctor, | Training needs self-assessment | -No name  -Experienced training librarians with input | Survey | Findings from a training needs analysis survey to support health |
|  |  |  |  |  |  |  | (Continued) |
| Supplementary material 7 Continued | | | | | | | |
| # | **Vancouver citation** | **Country/location**  **and Organisation** | **Workforce studied.** | **Objective of assessment**  **(Organisational, individual)** | **Assessment Tool’s name and methods of development** | **Methods used for the assessment** | **Tool: Availability** |
|  | research lifecycle. Health Info Libr J. 2020;37(2):118-27. | Darwin/Palmerston Katherine  Alice Springs Gove/Nhulunbuy  Tennant Creek  health department | Educator Manager,  Nurse or Midwife,  Policy Officer, Professional, Researcher, and Technical |  | from a Clinical Nurse Educator and the Library Director. Development of the survey was informed by the research lifecycle model proposed by Vaughan et al. (2013) and the literature investigating the education and training needs of health professionals |  | professionals across the research lifecycle - PubMed (nih.gov)  Page 9 |
| 10 | Bulawayo M, Silumbwe A, Munakampe MN, Mukumbuta N, Musabula J, Chewe M, et al. A needs assessment for postgraduate training in selected public health disciplines: evidence from health services organisations in Lusaka, Zambia. BMC Health Serv Res. 2020;20(1):1079. | Zambia  Lusaka  Public and private health service organisations | Representatives of health services organisations: regulatory authorities, research institutions, government ministries, insurance firms and other cooperating partners. | Training needs self-assessment  and organisational assessment | - No name  - The questions, developed after an extensive review of similar literature were piloted and refined before the commencement of data collection | Semi-structured questionnaire | https://static-content.springer.com/esm/art%3A10.1186%2Fs12913-020-05935-7/MediaObjects/12913_2020_5935_MOESM1_ESM.pdf |
|  |  |  |  |  |  |  | (Continued) |
|  |  |  |  |  |  |  |  |
| Supplementary material 7 Continued | | | | | | | |
| # | **Vancouver citation** | **Country/location**  **and Organisation** | **Workforce studied.** | **Objective of assessment**  **(Organisational, individual)** | **Assessment Tool’s name and methods of development** | **Methods used for the assessment** | **Tool: Availability** |
| 11 | Lee SA, Byth K, Gifford JA, Balasubramanian M, Fozzard CA, Skapetis T, et al. Assessment of Health Research Capacity in Western Sydney Local Health District (WSLHD): A Study on Medical, Nursing and Allied Health Professionals. J Multidiscip Healthc. 2020;13:153-63. | Australia  Sidney  Local Health District, | Medical  Nursing  Allied Health  Dentist  Role:  Clinician  Management and executive  Teaching and research | Training needs self-assessment | -The research capacity and culture (RCC) tool.  The methods to validate RCC tool in public health workforce (Validation of the research capacity and culture (RCC) tool: measuring RCC at individual, team and organisation levels - PubMed (nih.gov)) | Survey | No |
| 12 | Bogaert K, Castrucci BC, Gould E, Sellers K, Leider JP, Whang C, et al. The Public Health Workforce Interests and Needs Survey (PH WINS 2017): An Expanded Perspective on the State Health Agency Workforce. J Public Health Manag Pract. 2019;25 Suppl 2, Public Health Workforce Interests and Needs Survey 2017:S16-S25. | USA  State Health Agency Workforce (SHA-CO) | The public health workforce. | Training needs self-assessment | Authors selected 2017 PH WINS.  PH WINS ‘Methods: Leider JP, Bharthapudi  K, Pineau V, Liu L, Harper E. The Methods Behind PH WINS. J Public Health Manag Pract. 2015;21 Suppl 6:S28-35. | Survey | Tool: Public Health Workforce Interests and Needs Survey - State (debeaumont.org)  Codebook  PH-WINS-2017-codebook_annotated_externalresearchers.pdf (debeaumont.org) |
|  |  |  |  |  |  |  | (Continued) |
| Supplementary material 7 Continued | | | | | | | |
| # | **Vancouver citation** | **Country/location**  **and Organisation** | **Workforce studied.** | **Objective of assessment**  **(Organisational, individual)** | **Assessment Tool’s name and methods of development** | **Methods used for the assessment** | **Tool: Availability** |
|  |  |  |  |  | The Methods Behind PH WINS - PubMed (nih.gov) |  |  |
| 13 | O'Meara MG, Sobelson RK, Trigoso SM, Kramer RE, McNaughton C, Smartis RJ, et al. Ensuring a competent public health responder workforce: The CDC experience. J Emerg Manag. 2019;17(3):199-209. | USA  Atlanta  CDC | CDC full-time staff who had served in an agency activation, deployment, and/or exercise | organisational assessment | - no name  - the authors did not provide how the three questions were created. | -interviews and focus groups (FGs)  -in-depth interviews  -brief interviews | Ensuring a competent public health responder workforce: The CDC experience (nih.gov)  Page 3 |
| 14 | McKeever, J.Leider, J. P.Alford, A. A.Evans, D. Regional Training Needs Assessment: A First Look at High-Priority Training Needs Across the United States by Region. J Public Health  Manag Pract. 2019;25 Suppl 2, Public Health Workforce Interests and Needs Survey 2017: S166-S176 | USA  State health agency-central office and local health department | Public health workforce working in the state health agency-central office and local health department, | Training needs self-assessment | Authors selected 2017 PH WINS.  PH WINS ‘Methods: Leider JP, Bharthapudi K, Pineau V, Liu L, Harper E. The Methods Behind PH WINS. J  Public Health Manag Pract. 2015;21 Suppl 6:S28-35.  The Methods Behind PH WINS - PubMed (nih.gov) | Survey | Tool: Public Health Workforce Interests and Needs Survey - State (debeaumont.org)  Codebook  PH-WINS-2017-codebook_annotated_externalresearchers.pdf (debeaumont.org) |
|  |  |  |  |  |  |  | (Continued) |
|  |  |  |  |  |  |  |  |
| Supplementary material 7 Continued | | | | | | | |
| # | **Vancouver citation** | **Country/location**  **and Organisation** | **Workforce studied.** | **Objective of assessment**  **(Organisational, individual)** | **Assessment Tool’s name and methods of development** | **Methods used for the assessment** | **Tool: Availability** |
| 15 | Bogaert K, Castrucci BC, Gould E, Rider N, Whang C, Corcoran E. Top Training Needs of the Governmental Public Health Workforce. J Public Health Manag Pract. 2019;25 Suppl 2, Public Health Workforce Interests and Needs Survey 2017:S134-S44. | USA  State and local health departments | Governmental public health workforce | Training needs self-assessment | Authors selected 2017 PH WINS.  PH WINS ‘Methods: Leider JP, Bharthapudi K, Pineau V, Liu L, Harper E. The Methods Behind PH WINS. J Public Health Manag Pract. 2015;21 Suppl 6:S28-35.  The Methods Behind PH WINS - PubMed (nih.gov) | Survey | Tool: Public Health Workforce Interests and Needs Survey - State (debeaumont.org)  Codebook  PH-WINS-2017-codebook_annotated_externalresearchers.pdf (debeaumont.org) |
| 16 | Yeager, V. A.Wisniewski, J. M. Chapple-McGruder, T.Castrucci, B. C.Gould, E. Public Health Workforce Self-Identified Training Needs by Jurisdiction and Job Type. J Public Health Manag Pract.2019; 25 (2):181-190 | USA  State and local public health agencies | public health employees in state and local public health agencies | Training needs self-assessment | Authors selected 2014 PH WINS  PH WINS ‘Methods: Leider JP, Bharthapudi K, Pineau V, Liu L, Harper E. The Methods Behind PH WINS. J | Survey | https://debeaumont.org/wp-content/uploads/2019/06/PH-WINS-2014-instrument.pdf |
|  |  |  |  |  |  |  | (Continued) |
| Supplementary material 7 Continued | | | | | | | |
| # | **Vancouver citation** | **Country/location**  **and Organisation** | **Workforce studied.** | **Objective of assessment**  **(Organisational, individual)** | **Assessment Tool’s name and methods of development** | **Methods used for the assessment** | **Tool: Availability** |
|  |  |  |  |  | Public Health Manag Pract. 2015;21 Suppl 6:S28-35.  The Methods Behind PH WINS - PubMed (nih.gov) |  |  |
| 17 | Arogundade, L. Akinwumi, T. Molemodile, S. Nwaononiwu, E. Ezika, J. Yau, I , et al. Lessons from a training needs assessment to strengthen the capacity of routine immunization service providers in Nigeria. BMC Health Serv Res.2019;19 (1):664. | Nigeria  3 Nigerian states Bauchi,  Niger, and  Rivers  College of Medical Sciences, Schools of Health Technology, School of Nursing and Midwifery. | -health workers offering immunization services. | -Survey 1 and 2: Training needs self-assessment  -Focus group: organisational assessment. | Strengthening Training for EPI & PHC in Nigeria (STEP-IN)  Structured data collection tools in survey format were specifically developed by the team.  -the tools were pretested and refined before deployment | -Survey 1 and 2  -Focus group | Survey 1: Backup_of_STEP IN PRoject 1 (springer.com)  Focus Group: https://static-content.springer.com/esm/art%3A10.1186%2Fs12913-019-4514-2/MediaObjects/12913_2019_4514_MOESM3_ESM.docx  Survey 2: step 5 (springer.com) |
| 18 | Joly, B. M.Coronado, F.Bickford, B. C.Leider, J. P.Alford, A.McKeever, J. A Review of Public Health Training Needs Assessment Approaches: Opportunities to | USA  Regional Public Health Training Centers. | Workforce capacity required to ensure effective delivery of essential public health service | Training needs self-assessment  and organisational assessment | - No name  -Authors conducted a review | Survey (this is an example) | jphmp_2017_12_07_joly_1700337_sdc1.docx (live.com) |
|  |  |  |  |  |  |  | (Continued) |
|  |  |  |  |  |  |  |  |
| Supplementary material 7 Continued | | | | | | | |
| # | **Vancouver citation** | **Country/location**  **and Organisation** | **Workforce studied.** | **Objective of assessment**  **(Organisational, individual)** | **Assessment Tool’s name and methods of development** | **Methods used for the assessment** | **Tool: Availability** |
|  | Move Forward. J Public Health Manag Pract. 2018; 24 (6): 571-577 |  |  |  |  |  |  |
| 19 | Mid-Atlantic Regional Public Health Training Center (MAR-PHTC). Public Health Training Needs Assessment: Report on Maryland Department of Health and Mental Hygiene.2017. Available from Training Needs Assessment Report - Maryland DHMH.pdf Accessed on 9^th^ July 2023 | USA  Region’s public health system. | The workforce to deliver public health services | Training needs self-assessment | No name  The evaluation team drafted the instruments. | -Qualitative interviews  - Two surveys  - The generic tool (questionnaire) | Tool not available. |
| 20 | Chapple-McGruder, T.Leider, J. P. Beck, A. J.Castrucci, B. C.Harper, E. Sellers, K. Examining state health agency epidemiologists and their training needs. Ann Epidemiol. 2017;27(2): 83-88 | USA  State  Health Agencies | Epidemiology workforce | Training needs self-assessment | 2014 PH WINS.  The authors selected this tool.  PH WINS ‘Methods: Leider JP, Bharthapudi K, Pineau V, Liu L, Harper E. The Methods Behind PH WINS. J Public Health Manag Pract. 2015;21 Suppl 6:S28-35. | Surveys | https://debeaumont.org/wp-content/uploads/2019/06/PH-WINS-2014-instrument.pdf |
|  |  |  |  |  |  |  | (Continued) |
| Supplementary material 7 Continued | | | | | | | |
| # | **Vancouver citation** | **Country/location**  **and Organisation** | **Workforce studied.** | **Objective of assessment**  **(Organisational, individual)** | **Assessment Tool’s name and methods of development** | **Methods used for the assessment** | **Tool: Availability** |
|  |  |  |  |  | The Methods Behind PH WINS - PubMed (nih.gov) |  |  |
| 21 | Kornfeld, J.Sznol, J.Lee, D. Characterizing the Business Skills of the Public Health Workforce: Practical Implications From the Public Health Workforce Interests and Needs Survey (PH WINS). J Public Health Manag Pract. 2015; 21 Suppl 6: S159-67 | USA  State Health Agency | State health agency employees | Training needs self-assessment | 2014 PH WINS.  The authors selected this tool.  PH WINS ‘Methods: Leider JP, Bharthapudi K, Pineau V, Liu L, Harper E. The Methods Behind PH WINS. J Public Health Manag Pract. 2015;21 Suppl 6:S28-35.  The Methods Behind PH WINS - PubMed (nih.gov) | Survey | https://debeaumont.org/wp-content/uploads/2019/06/PH-WINS-2014-instrument.pdf |
| 22 | Ye, J. Leep, C.Robin, N.Newman, S. Perception of Workforce Skills Needed Among Public Health Professionals in Local Health Departments: Staff Versus Top Executives. J Public Health Manag Pract.2015; 21 Suppl 6:S151-8 | USA  Arkansas,  Georgia, Mississippi,  South Carolina, Vermont, Washington, and Wisconsin | Top executives and staff. | Training needs self-assessment | 2014 PH WINS  2015 FoC  Authors selected these tools.  PH WINS’ Methods: Leider JP, Bharthapudi K, Pineau V, Liu L, Harper E. The Methods | Survey | -PH WINS: https://debeaumont.org/wp-content/uploads/2019/06/PH-WINS-2014-instrument.pdf |
|  |  |  |  |  |  |  | (Continued) |
| Supplementary material 7 Continued | | | | | | | |
| # | **Vancouver citation** | **Country/location**  **and Organisation** | **Workforce studied.** | **Objective of assessment**  **(Organisational, individual)** | **Assessment Tool’s name and methods of development** | **Methods used for the assessment** | **Tool: Availability** |
|  |  | -State Health Agencies;  -LHD members of the Big City Health Coalition.  -LHDs in a 7-state local pilot |  |  | Behind PH WINS. J Public Health Manag Pract. 2015;21 Suppl 6:S28-35.  The Methods Behind PH WINS - PubMed (nih.gov) |  | - 2015 FoC  :https://www.naccho.org/uploads/downloadable-resources/2015-Forces-of-Change-Survey.pdf  Codebook: https://www.naccho.org/uploads/downloadable-resources/2015-Forces-of-Change-Survey_Codebook.pdf |
| 23 | Dixon, B. E.McFarlane, T. D.Dearth, S. Grannis, S. J.Gibson, P. J. Characterizing Informatics Roles and Needs of Public Health Workers: Results From the Public Health Workforce Interests and Needs Survey. J Public Health Manag Pract.2015; 21 Suppl 6: S130-40 | USA  -State health Agencies,  -members of the Big City Health Coalition;  -Local Health Department. | Informatics; information technology (IT); clinical and laboratory; and other public health science specialists working in state health agencies | Training needs self-assessment | 2014 PH WINS.  Authors selected this tool.  PH WINS ‘Methods: Leider JP, Bharthapudi K, Pineau V, Liu L, Harper E. The Methods Behind PH WINS. J Public Health Manag Pract. 2015;21 Suppl 6:S28-35. | Survey | https://debeaumont.org/wp-content/uploads/2019/06/PH-WINS-2014-instrument.pdf |
|  |  |  |  |  |  |  | (Continued) |
| Supplementary material 7 Continued | | | | | | | |
| # | **Vancouver citation** | **Country/location**  **and Organisation** | **Workforce studied.** | **Objective of assessment**  **(Organisational, individual)** | **Assessment Tool’s name and methods of development** | **Methods used for the assessment** | **Tool: Availability** |
|  |  |  |  |  | The Methods Behind PH WINS - PubMed (nih.gov) |  |  |
| 24 | Sellers, K.Leider, J. P.Harper, E. Castrucci, B. C.Bharthapudi, K. Liss-Levinson, R, et al. The Public Health Workforce Interests and Needs Survey: The First National Survey of State Health Agency Employees. J Public Health Manag Pract. 2015; 21 Suppl 6: S13-27. | USA  State Health Agency | -Public health practitioners, policy makers, and researchers  - Public health workforce— those who influence the entire public health system by cultivating and curating the necessary inputs and processes through which population outcomes are achieved | Training needs self-assessment | PH WINS 2014.  Authors selected this tool.  PH WINS ‘Methods: Leider JP, Bharthapudi K, Pineau V, Liu L, Harper E. The Methods Behind PH WINS. J Public Health Manag Pract. 2015;21 Suppl 6:S28-35  The Methods Behind PH WINS - PubMed (nih.gov) | Survey | https://debeaumont.org/wp-content/uploads/2019/06/PH-WINS-2014-instrument.pdf |
| 25 | Bloomington Public Health .Staff Training Needs Assessment Bloomington Public Health. 2014.Available from: BPH Training Needs Assessment | USA  Bloomington/Minneapolis.  Bloomington Public Health. | Bloomington Public Health staff and leadership | Training needs self-assessment | The Council on Linkages Core Competencies for Public Health Professionals assessments | Questionnaire | BPH Training Needs Assessment Report (naccho.org) |
|  |  |  |  |  |  |  | (Continued) |
| Supplementary material 7 Continued | | | | | | | |
| # | **Vancouver citation** | **Country/location**  **and Organisation** | **Workforce studied.** | **Objective of assessment**  **(Organisational, individual)** | **Assessment Tool’s name and methods of development** | **Methods used for the assessment** | **Tool: Availability** |
|  | Report (naccho.org) Accessed on 9^th^ July 2023 |  |  |  | Authors selected this tool.  The Council on Linkages Between Academia and Public Health Practice. Core Competencies for Public Health Professionals. Available from:  2021 Core Competencies for Public Health Professionals (phf.org) |  |  |
| 26 | Adkoli, B. V.Al-Umran, K. U. Al-Sheikh, M. H.Deepak, K. K. Innovative method of needs assessment for faculty development programs in a Gulf medical school. Educ Health (Abingdon).2010;23(3): 389. | Saudi Arabia  Damman  University of Dammam | Faculty members (The Medical Education Unit of the College of Medicine) | Training needs self-assessment | Needs Assessment Survey of Faculty Development Programs at College of Medicine, University of Dammam, Saudi Arabia – 2009  A questionnaire was designed, pilot-tested and administered to all faculty members | Survey | Innovative method of needs assessment for faculty development programs in a Gulf medical school - PubMed (nih.gov)  Appendix |
| 27 | Hites, L. S. Lafreniere, A. V. Wingate, M. S. Anderson, A. C. Ginter, P. M.Santacaterina, L, et al. Expanding the public health | USA | Public health emergency responders | Training needs self-assessment | The matrix does not have name. | Objective-competency matrix tool. | Expanding the public health emergency preparedness competency set to meet specialized |
|  |  |  |  |  |  |  | (Continued) |
| Supplementary material 7 Continued | | | | | | | |
| # | **Vancouver citation** | **Country/location**  **and Organisation** | **Workforce studied.** | **Objective of assessment**  **(Organisational, individual)** | **Assessment Tool’s name and methods of development** | **Methods used for the assessment** | **Tool: Availability** |
|  | emergency preparedness competency set to meet specialized local and evolving national needs: a needs assessment and training approach. J Public Health Manag Pract.2007; 13(5): 497-505 | South Central Center for Public Health Preparedness |  |  | Methods: a modified Q-sort and repeating the modified Q-sort |  | local and evolving national needs: a needs assessment and training approach - PubMed (nih.gov)  Table 4. |
| 28 | Dato, V. M. Potter, M. A. Fertman, C. I. Training readiness of public health agencies: a framework for assessment. J Public Health Manag Pract.2001; 7(4): 91-5. | USA  Cohort of scholars in the Northeast Regional Public Health Leadership  Institute ( NEPHLI) were selected to create a framework for the State and local agencies | Employees from state and local agencies | Training needs self-assessment | No name.  Learning organization theory was used.  a workshop was done. | Questionnaire | Training readiness of public health agencies: a framework for assessment - PubMed (nih.gov)  Pages 93 and 94 |
| 29 | Ruck, N. F.Hafeez, M. Manzur, S. Nasir, F. Rivers, K. Prozesky, D. Assessing management training needs: a study in the Punjab health services, Pakistan. J Health Popul Dev Ctries.1999;2 (1): 78-87 | Pakistan  Province of Punjab.  Provincial Health Development Center | District managers | Training needs self-assessment  and organisational assessment | No name.  The authors did not provide a method section | “Phase 1: Semi-structured interviews.  -Phase 2: interviews.  - Phase 3: Focus group. | Phase 1 and 3:  Assessing management training needs: a study in the Punjab health services, Pakistan - PubMed (nih.gov)  Phase 2: tool not available |
|  |  |  |  |  |  |  | (Continued) |
| Supplementary material 7 Continued | | | | | | | |
| # | **Vancouver citation** | **Country/location**  **and Organisation** | **Workforce studied.** | **Objective of assessment**  **(Organisational, individual)** | **Assessment Tool’s name and methods of development** | **Methods used for the assessment** | **Tool: Availability** |
| **30** | **Western Region.** Public Health Training Center. Needs Assessment. Available from: Needs Assessment \| Western Region Public Health Training Center (arizona.edu) Accessed on 9^th^ July 2023 | USA  Arizona,  Nevada,  California,  Hawaii´s Pacific Islands  Western Region Public Health Training Center. | Public Health Workforce. | Training needs self-assessment | Self-assessment and Training Preferences Survey (PHCCSTPS)  Authors did not provide the methods | Survey | Microsoft Word - WRPHTC blank assessment report.docx (arizona.edu) |
| **31** | Academy for Educational Development. Support for Croatia’s non-governmental Sector CroNGO: Organizational Capacity Self- Assessment Tool Guidelines for Developing an Organizational Training Plan.  Available from: Organizational Capacity Self-Assessment Tool.pdf (ngoconnect.net) Accessed on 9^th^ July 2023 | Croatia.  Croatia's non-governmental sector. | NGO | Training needs self-assessment | Organizational Capacity Self-Assessment: CroNGO Training Component  Authors did not provide the methods | Questionnaire | Organizational Capacity Self-Assessment Tool.pdf (ngoconnect.net) |

| Supplementary material 8 List of interest: organisations that have created a framework that can be adapted into a tool: Training needs assessment tools for public health workforce at institutional and individual level - a review | | | | |
| --- | --- | --- | --- | --- |
| # | **Organisations** | **Country** | **Workforce studied** | **Vancouver citation** |
| 36 | American Nurses Association. | USA | Nurses | American Nurses Association. ANA LEADERSHIP COMPETENCY MODEL. 2018. Available from: 177626 ANA Leadership Booklet 01 (nursingworld.org) Accessed on 9^th^ July 2023 |
| 37 | American Organization of Nurse Executives. | USA | Nurses | American Organization of Nurse Executives. AONE Nurse Executive Competencies. 2015. Available from: https://www.aonl.org/sites/default/files/aone/nec.pdf Accessed on 9^th^ July 2023 |
| 38 | American Organization of Nurse Executives. | USA | Nurses | American Organization of Nurse Executives. AONE Nurse Executive Competencies. 2005. Available from: https://www.nurseleader.com/action/showPdf?pii=S1541-4612%2805%2900007-8 Accessed on 9^th^ July 2023 |

| Supplementary material 9 Tools identified in the review by handsearching: Training needs assessment tools for public health workforce at institutional and individual level - a review. | | | | | | |
| --- | --- | --- | --- | --- | --- | --- |
| # | **Organisations** | **Country** | **Workforce studied** | **Objective of assessment**  **(Organisational, individual)** | **Methods used for the assessment** | **Vancouver citation** |
| 32 | First Nations Health Managers Association | Canada | Health Managers | Training needs self-assessment | Questionnaire | First Nations Health Managers Association. The First Nations Health Manager Competency Framework Self-Assessment Tool. 2019. Available from: PowerPoint Presentation (fnhmaconference.ca) Accessed on 9^th^ July 2023 |
| 33 | National Association of County and City Health Officials. | USA | Local Health Departments | Training needs self-assessment | Survey | National Association of County and City Health Officials. 2018 Forces of Change. 2018. Available from: https://www.naccho.org/uploads/downloadable-resources/2018-Forces-of-Change_March-2018f.pdf Accessed on 9^th^ July 2023 |
| 34 | GOV.UK | UK | individuals who are pursuing, or wish to pursue a career in public health | Training needs self-assessment | Survey | GOV.UK. Public health skills and knowledge framework: tools and guidance. 2018.Available from: PHSKF: self-assessment tool. Accessed on 9^th^ July 2023 |
| 35 | National Health System | UK | Clinicians | Training needs self-assessment | Survey | NHS Leadership Academic. Clinical Leadership Competency Framework: Self-assessment tool. 2012. Available from: CLCF Self Assessment_Layout 1 (leadershipacademy.nhs.uk) Accessed on 9^th^ July 2023 |
